# Charge localization in a diamine cation provides a test of energy functionals and self-interaction correction

**DOI:** 10.1038/ncomms11013

**Published:** 2016-03-16

**Authors:** Xinxin Cheng, Yao Zhang, Elvar Jónsson, Hannes Jónsson, Peter M. Weber

**Affiliations:** 1 Department of Chemistry, Brown University, 324 Brook Street, Providence, Rhode Island 02912, USA; 2 COMP, Department of Applied Physics, Aalto University, FIN-00076 Espoo, Finland; 3 Faculty of Physical Sciences, VR-III, University of Iceland, 107 Reykjavík, Iceland

## Abstract

Density functional theory (DFT) is widely applied in calculations of molecules and materials. Yet, it suffers from a well-known over-emphasis on charge delocalization arising from self-interaction error that destabilizes localized states. Here, using the symmetric diamine *N*,*N*′-dimethylpiperazine as a model, we have experimentally determined the relative energy of a state with positive charge localized on one of the two nitrogen atoms, and a state with positive charge delocalized over both nitrogen atoms. The charge-localized state was found to be 0.33 (0.04) eV higher in energy than the charge-delocalized state. This provides an important test of theoretical approaches to electronic structure calculations. Calculations with all DFT functionals commonly used today, including hybrid functionals with exact exchange, fail to predict a stable charge-localized state. However, the application of an explicit self-interaction correction to a semi-local functional identifies both states and gives relative energy in excellent agreement with both experiment and CCSD(T) calculations.

## Abstract (web summary)

Density functional theory is widely used throughout the chemical sciences, but suffers from over-emphasis on charge delocalisation. Here, the authors experimentally probe the energies of two states of a diamine cation and show how a self-interaction correction allows for the accurate prediction of both states.

## Graphical abstract

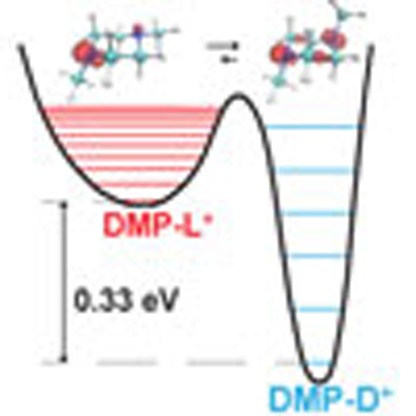

Charge transfer (CT) between two or more charge centres and charge delocalization across separate parts of a molecular system are of great importance in many areas of chemistry as they often define molecular structure and reactivity^1^,^2^,^3^. Their relevance further extends to biological polymers such as DNA and to light-harvesting processes using artificial photosynthesis^3^,^4^,^5^,^6^,^7^. Localized electronic states and separation of charges are, furthermore, an important aspect of semiconductor devices and solar cells^8^,^9^,^10^,^11^,^12^. Yet, the calculation of charge localization and charge delocalization is challenging, especially for computational methods that are applicable to extended systems.

The commonly used Kohn–Sham density functional theory (DFT) functionals are known to suffer from bias towards delocalized states because of self-interaction error^13^,^14^,^15^. Hartree–Fock (HF), on the other hand, is overly biased towards localized states. Hybrid functionals that mix the two approaches are widely used in all branches of chemistry and are increasingly used in condensed matter calculations, but the balance between localized and delocalized states depends strongly on the proportions used in the mixing. This mixing ratio is sometimes treated as an empirical, system-dependent parameter. A more fundamental, parameter-free approach is, however, needed to accurately predict the delicate balance between localized and delocalized electronic states, especially in extended systems. Over 30 years ago, an explicit self-interaction correction approach was proposed by Perdew and Zunger (PZ-SIC)^16^. Although it was applied early on in studies of the electronic structure of molecules (see, for example, ref. 17), it has not become a commonly used approach. A variational, self-consistent implementation of this PZ-SIC using complex valued orbitals has recently been applied successfully in studies of molecules and solids, including Rydberg excited states^18^,^19^,^20^. A discussion of the self-interaction error and our implementation of PZ-SIC can be found in Supplementary Note 1. We show here that although all the commonly used DFT functionals, including hybrid functionals, fail to produce the localized charge state of the system studied, calculations using PZ-SIC give results in excellent agreement with the experimentally determined relative energy of a localized and delocalized electronic state.

To achieve this comparison, experimental benchmarks for charge-localized and charge-delocalized states are required. This is challenging because in most cases electronic systems exhibit just one particular charge distribution, making comparison between states with different charge distributions difficult. In a recent study, however, we have found that in *N,N'*-dimethylpiperazine (DMP), both a charge-localized state and a charge-delocalized state can be observed^21^. DMP has previously served as a prototype for exploring electron lone pair interactions and CT between nitrogen atoms^22^,^23^,^24^. In its cationic state, the positive charge can be localized on one of the nitrogen atoms (DMP-L^+^), or delocalized over the two equivalent nitrogen atoms (DMP-D^+^). The charge-localized ion DMP-L^+^, however, has not been observed until the recent ultrafast time-resolved experiment^21^. It is possible to identify the different charge states because they have distinct spectral and temporal signatures. Upon optical excitation, the charge-localized state is initially generated. A subsequent CT then leads to the charge-delocalized state. This discovery of two states with distinct charge distributions has now laid the foundation for an experimental approach for measuring their relative energy. The approach is based on photoionization from Rydberg states, whose binding energies have been found to be remarkably dependent on the nuclear arrangements and the charge distribution of the molecular ion core. Further, the Rydberg electron-binding energy is independent of a molecule's internal energy, making it ideally suited for the exploration of high-energy charge states that are populated when the molecules are highly energized^25^,^26^,^27^,^28^. Because the Rydberg electron has a small effect on the bonding and molecular structure, the configuration of the molecular ion cores of Rydberg states closely resemble those of the cationic states. The binding energies of the Rydberg states, therefore, yield information about the charge-localized and charge-delocalized cationic states.

The relative energy of a charge-localized state and a charge-delocalized state of a given molecule has not been determined previously, as far as we know, even though it is a critically important parameter for calibrating theoretical approaches. In the current study, the relative energy was determined using a newly devised experimental approach that takes advantage of an equilibrium established on a picosecond time scale upon excitation to the Rydberg states. Calculations using conventional DFT, self-interaction-corrected DFT and *ab initio* methods were tested against the experimental measurements. We aim to evaluate the capability of each method to properly describe the charge localization and delocalization in the ground electronic state of the molecular cation.

## Results

### Experimental determination of the relative energy

To measure the energy of the cationic state, we first experimentally determined the relative energy of 3sD and 3sL Rydberg states with the charge-delocalized ion core DMP-D^+^ and the charge-localized ion core DMP-L^+^, respectively, by measuring the equilibrium composition of the Rydberg states as a function of the excitation energy. As shown in Supplementary Fig. 1, because the binding energy, that is, the energy difference between the cationic state and the Rydberg state, is measured in the experiment, the relative energy of the cationic states is known once the relative energy of the 3s Rydberg states is known. DMP was excited from its ground state to the 3p or the 3s Rydberg state using pump photons with wavelengths tuned in the range of 193.0–240.8 nm. The probe photon monitored the time-dependent dynamics by ionizing the Rydberg-excited molecules. The kinetic energy of the ejected photoelectrons was measured to determine the binding energy of the Rydberg states. The 3p state, which was reached with the shorter wavelengths, decayed by internal conversion into 3s within several hundred femtoseconds. Details of the experimental setup and parameters are given in Supplementary Note 2. As we have shown previously, upon optical excitation to the localized charge state, an equilibrium between the localized and delocalized states is reached after several picoseconds^21^.

The complete set of the time-resolved photoelectron spectra at several pump wavelengths are given in Supplementary Fig. 2. Because the energy is conserved in internal conversion, pump photons of different wavelengths deposit different amounts of energy into the vibrational manifolds. For pump wavelengths between 193.0 and 240.8 nm, the molecule has effective vibrational temperature between 565 and 980 K after relaxation, assuming the energy is distributed across all vibrational modes. The detailed calculation of the effective temperature is discussed in the Supplementary Note 6.

**Figure 1:**
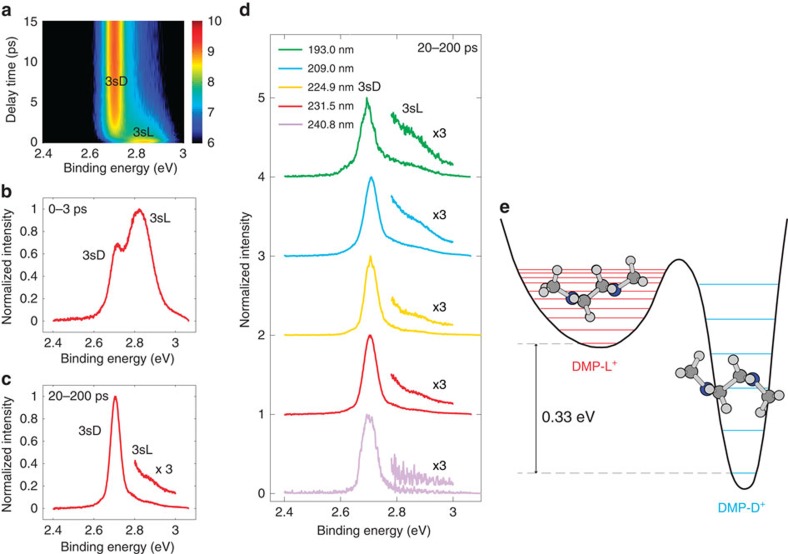
Photoelectron spectra of DMP. (**a**) The time-resolved spectrum of DMP with 231.5 nm pump photon. The colour bar represents the logarithmic intensity scale. (**b**) The 0–3 ps time-integrated spectrum of **a**. (**c**) The 20–200 ps time-integrated spectrum of **a**. (**d**) The 20–200 ps time-integrated spectra of DMP at five selected pump wavelengths. The relative populations of the charge-localized (3sL) and the charge-delocalized (3sD) states can be determined as a function of temperature from this data, thus providing an estimate of the relative energy of the two states, which turns out to be 0.33 eV. (**e**) A schematic cut of the potential energy surface for DMP^+^. The red and blue lines illustrate the vibrational states of DMP-L^+^ and DMP-D^+^, respectively.

Two 3s peaks (Fig. 1), located at 2.70 (0.03) eV and at 2.81 (0.04) eV, are assigned to 3sD and 3sL, respectively^21^. The 3sL dominates at short delay times (Fig. 1a,b), whereas 3sD dominates at longer delay times (Fig. 1a,c,d), indicating an early population in 3sL and a lower energy for 3sD. As the temperature decreases from 980 to 565 K with the pump wavelength increasing from 193.0 nm to 240.8 nm, the intensity of 3sL decreases almost to the baseline in the spectra with the molecules at equilibrium (Fig. 1d)

Because the change of the Gibbs free energy (Δ*G*) scales linearly with the natural logarithm of the equilibrium constant (*K*), the enthalpy (Δ*H*) and entropy (Δ*S*) change in the transition can be determined from the logarithm of the equilibrium constant as a function of inverse temperature:


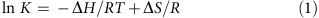


where *R* is the gas constant and *T* is the temperature.

**Figure 2:**
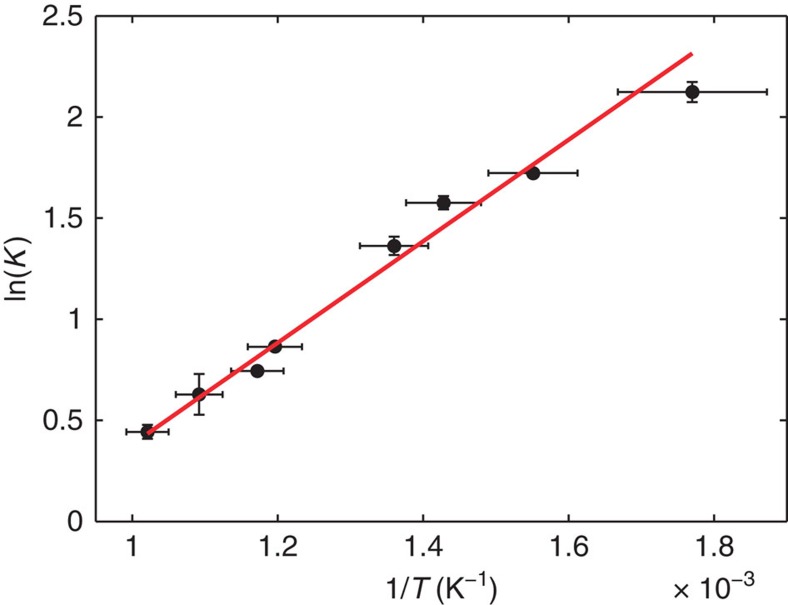
Temperature dependence of the equilibrium constants. Measured values of the equilibrium constant for the 3sL to 3sD states of the DMP molecule are shown as a function of reciprocal temperature estimated from the photon energy. The red line shows a linear best fit providing an estimate of the energy and entropy difference between the two states.

The spectra at each point in time were fitted using two Lorentzians with variable peak centres to derive the equilibrium constants in the 3s states, as described in Supplementary Notes 4 and 5. Details of the fits are shown in Supplementary Notes 4 and 5, Supplementary Figs 3 and 4 and Supplementary Tables 1 and 2. The results are shown in Fig. 2. The logarithm of the equilibrium constant is indeed found to depend approximately linearly on the estimated reciprocal temperature. A fit using equation (1) gives −20.9 (3.7) kJ mol^−1^ or −0.22 (0.04) eV and −17.7 (4.6) J K^−1^ mol^−1^ for Δ*H* and Δ*S* of the transition from 3sL to 3sD. Because the binding energy difference between 3sL and 3sD is 0.11 (0.01) eV, the energy of the DMP-L^+^ ion is determined to be 0.33 (0.04) eV higher than that of the DMP-D^+^ ion. A schematic cut through the energy surface, deduced from these measurements, is shown in Fig. 1e.

### Test of theoretical methods

The ability of various theoretical approaches to describe the charge localized and delocalized states can now be assessed by comparison with these experimental results. First, calculations were carried out to determine the optimal molecular geometries of the two states. The DMP-L^+^ and DMP-D^+^ ion structures were optimized with the Gaussian 09 (refs 29, 30) and NWChem software^31^ at various levels of theory including HF, MP2 (Møller–Plesset perturbation theory, truncated at the second order), DFT with all the commonly used functionals (complete list available in Supplementary Note 2), and CCSD (coupled cluster method with single and double excitations). Unless specified otherwise, the aug-cc-pVDZ basis set was used in the Rydberg state calculations and the cc-pVTZ basis set in the cation calculations^32^,^33^. The cation structures were also optimized using PZ-SIC with the GPAW software^34^,^35^,^36^, where a real space grid over a cubic simulation cell of 20 Å side length and 0.13 Å mesh was used. The PZ-SIC was applied to the PBE semi-local functional. Although in the neutral molecule each nitrogen atom assumes a pyramidal structure with the methyl group in equatorial position, the nitrogen becomes pseudo-axial and pseudo-planar in the cation. The Cartesian coordinates of selected optimized structures are listed in Supplementary Tables 4 and 5.

**Figure 3:**
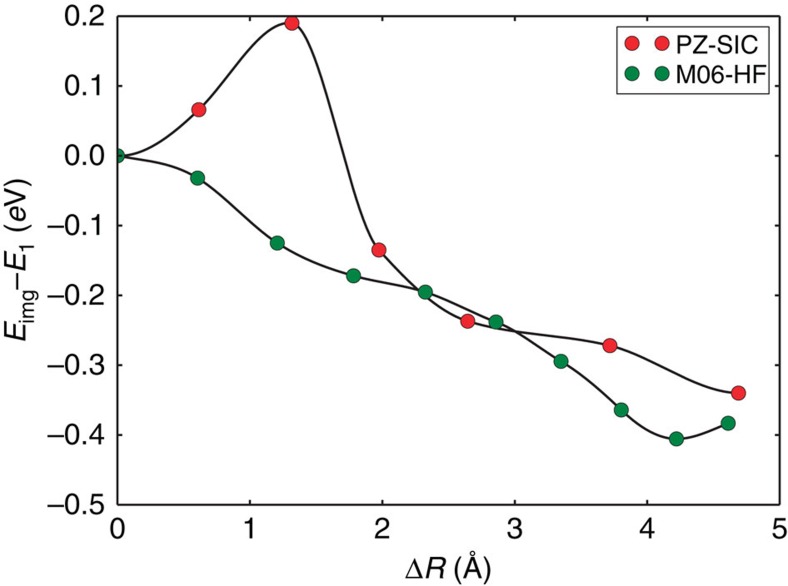
Calculated minimum energy path between the localized and delocalized state of the DMP cation. The energy of images, *E*_img_, in the nudged elastic band calculations is given with respect to the energy of the localized state, *E*_1,_ as a function of the accumulated displacement of the atoms, Δ*R*. The red dots show results of a PZ-SIC calculation where a barrier of 0.2 eV separates the metastable, localized state from the delocalized state. The green dots show results of calculations using the M06-HF functional where MP2 optimized structures are used for the end points. In the M06-HF calculations, the energy barrier is not present and a structure optimization starting from the localized state converges on the delocalized state. Similar results were obtained for all other commonly used DFT functionals.

DFT calculations with any one of the available hybrid functionals implemented in the Gaussian 09 software failed to provide a stable localized state, except for the BHandHLYP functional that has previously been found to describe CT interactions well^37^ while giving generally poor results for other molecular properties such as total energy (and therefore not commonly used)^38^. When DFT calculations were started from MP2 or HF-optimized structures for the localized state, the minimization of the energy resulted in a conversion to the charge delocalized state. Most surprisingly, the M06-HF functional, which contains 100% HF exchange and is therefore widely deemed to be particularly appropriate for CT^39^,^40^,^41^, also fails to localize the charge in this case. However, the PZ-SIC calculation gives a stable localized state with similar structure as that obtained from MP2 and CCSD. The bond lengths are typically 0.02–0.04 Å shorter than those obtained from MP2 and CCSD. The minimum energy path between the two states calculated using the nudged elastic band method^42^ and the PZ-SIC as well as the M06-HF functional is shown in Fig. 3. An energy barrier of 0.2 eV for the transition from the localized to the delocalized state is obtained with PZ-SIC, whereas no barrier is obtained in the M06-HF calculations.

Table 1 lists the calculated energy difference between the optimized DMP-L^+^ and DMP-D^+^ structures obtained using HF, MP2, CCSD, DFT with selected functionals and PZ-SIC. CCSD(T) calculations were carried out to obtain the single-point energy for each one of these structures. Although calculations using HF, MP2 and CCSD produce both the DMP-L^+^ and the DMP-D^+^ states, the relative energy of these states is poorly estimated, especially by HF, which gives lower energy for the localized state than the delocalized state. Single-point CCSD(T) calculations using the MP2 or CCSD geometries give a relative energy in good agreement with the experimental measurements. The PZ-SIC calculation also yields a relative energy that is close to the experimental results, see Table 1 and Supplementary Table 3. Supplementary Note 7 also reports a satisfactory agreement of the experimental and computed entropy differences.

The close agreement between the relative energy obtained from the high-level CCSD(T) calculations and the experimental results confirms the validity of the interpretation of the experimental data. To further cement the correspondence of experimental and computational results, we have calculated the Rydberg electron-binding energy with the optimized DMP-L^+^ and DMP-D^+^ structures using the equation of motion CCSD. For comparison, the binding energy was also calculated using PZ-SIC, which has previously been shown to give good estimates of Rydberg binding energy of both molecules and molecular clusters^18^,^27^,^28^,^43^. The total energy of the Rydberg excited states using PZ-SIC was obtained using the delta self-consistent field method^44^ and the binding energy obtained by subtracting the total energy of the excited state from that of the ion. As listed in Table 2, the calculated binding energy is in good agreement with the experimentally measured values for both the DMP-L^+^ and DMP-D^+^ structures, supporting the assignment of the observed spectroscopic features.

**Figure 4:**
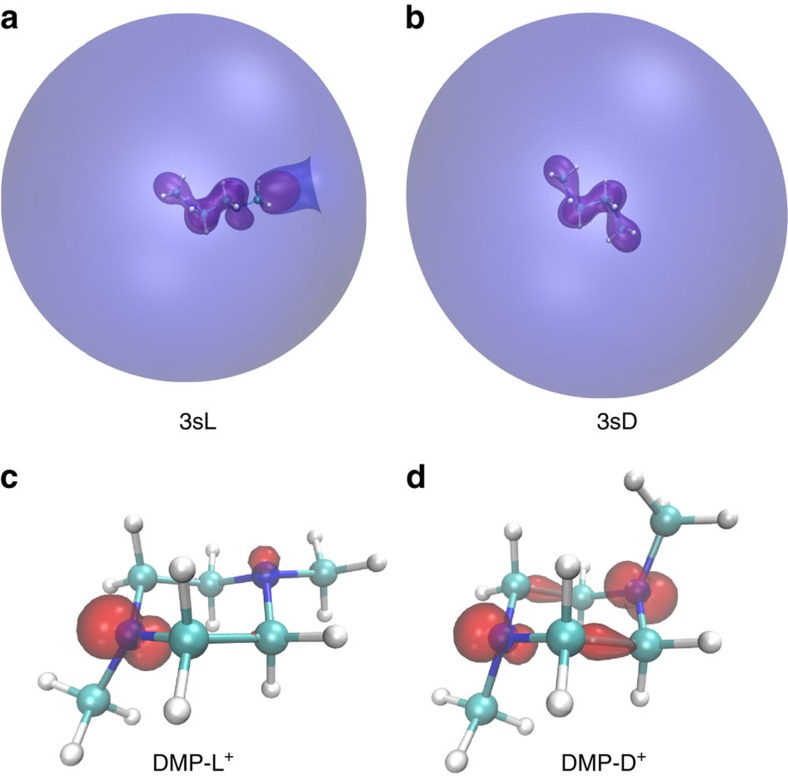
The Rydberg orbitals and the associated spin densities. (**a**,**b**) Calculated 3sL and 3sD Rydberg orbitals, respectively, rendered at 0.001 Å^−3/2^ isovalues. (**c**,**d**) Calculated spin density of the DMP-L^+^ and DMP-D^+^ ion, respectively, at isovalue of 0.2 electron per Å^−3^.

The Rydberg orbitals and the associated spin densities are shown in Fig. 4. The 3sL Rydberg orbital (Fig. 4a) anchors on the planar nitrogen, whereas the 3sD Rydberg orbital (Fig. 4b) is centred symmetrically between the two nitrogen atoms. Both orbitals are extended and comprise the whole molecule, as expected. The spin densities (shown in Fig. 3c,d), which were generated by subtracting the spin-down density from the spin-up density, illustrate the charge distributions of the localized and delocalized cations. The charge is localized on the planar nitrogen in the DMP-L^+^ (Fig. 3c) but delocalized between the two nitrogen atoms in DMP-D^+^ (Fig. 3d). Intriguingly, there is also net spin density between the two intermediate C-atoms, indicating a through-bond-interaction^22^ in the charge-delocalized ion.

## Discussion

The present study advances experimentally the state-of-the-art in exploring charge localization and delocalization in systems with multiple charge centres. The wavelength-dependent Rydberg electron-binding energy spectroscopy enables us to experimentally determine the energy and entropy change of the CT reaction. It is the experimental tool of choice to probe the CT process in the presence of large vibrational energy, as is needed to observe the higher-energy state before CT. Indeed, previous time-resolved spectroscopic studies on DMP radical cations prepared by a photo-induced electron transfer technique only observed the DMP-D^+^ ion^23^,^24^ possibly because of the low temperature in the system. Together with the binding energy information obtained from the spectra, the present experiment creates an important benchmark to evaluate various theoretical approaches.

The MP2 and CCSD methods are found to give good estimates of the molecular structure as the single-point CCSD(T) calculation gives good agreement with the experimental binding energies of the 3sL and 3sD states as well as the relative energy of the two cation states. The computational effort of these methods, however, scales unfavourably with system size and they are thus limited to small systems. The computational effort of DFT calculations increases slower with size as *N*^3^, where *N* is the number of electrons, and is the only viable approach for many problems involving large molecules and condensed phase systems. Conventional DFT functionals are, however, found here not to predict the metastable localized state of DMP. The explicit inclusion of the self-interaction correction, as proposed by Perdew and Zunger, implemented in a variational and self-consistent way with complex-valued orbitals, can remedy these shortcomings of the DFT approach. The PZ-SIC calculations give similar results for the binding energies of the 3sL and 3sD states as the equation of motion CCSD calculations and for the relative energies of the DMP-L^+^ and DMP-D^+^ states as the CCSD(T) calculations. The computational effort in this approach is larger than conventional DFT but still scales with size as *N*^3^. These results are expected to guide future improvements to energy functionals describing electronic systems.

## Additional information

**How to cite this article:** Cheng, X. *et al*. Charge localization in a diamine cation provides a test of energy functionals and self-interaction correction. *Nat. Commun.* 7:11013 doi: 10.1038/ncomms11013 (2016).

## Supplementary Material

Supplementary Information Supplementary Figures 1-4, Supplementary Tables 1-6, Supplementary Notes 1-7 and Supplementary References

## Figures and Tables

**Table 1:** RE of the DMP-L^+^ and DMP-D^+^ states obtained using various computational methods.

Method	RE (eV)
HF	−0.53
MP2	0.81
	
*DFT*	
B3LYP	—a
M06	— a
M06-2X	— a
M06-HF	— a
PBE0	— a
BHandHLYP	0.19
PZ-SIC	0.34
CCSD^b^	0.23
MP2_CCSD(T)-SP	0.39
CCSD_CCSD(T)-SP	0.38
Experiment	0.33 (0.04)

CCSD, coupled cluster method with single and double excitations; DFT, density functional theory; DMP, *N,N'*-dimethylpiperazine; HF, Hartree–Fock; PZ-SIC, Perdew and Zunger self-interaction correction; RE, relative energy.

Single-point energy calculations were carried out with the CCSD(T) method, in one case using a structure obtained with MP2 and in the other case using a structure obtained with CCSD.

Zero point energy correction has not been applied but an estimate based on ground vibrational states would reduce the calculated relative energy by 0.07 eV, see Supplementary Note 3. As the molecule is at high temperature, the full, ground vibrational state zero point correction is an overestimate.

^a^No value shown because the DMP-L^+^ state was not stable at this level of theory.

^b^The CCSD optimizations were carried out with the aug-cc-pVDZ basis set because calculations with the cc-pVTZ were found to be too demanding for our computational resources.

**Table 2:** Calculated Rydberg binding energy (in eV) of 3sD and 3sL states for MP2 optimized DMP-L^+^ and DMP-D^+^ structures using the EOM-CCSD and PZ-SIC methods.

Method	3sD	3sL
PZ-SIC	2.71	2.87
EOM-CCSD	2.65	2.72
Experiment	2.70 (0.03)	2.81 (0.04)

EOM-CCSD, equation of motion coupled cluster method with single and double excitations; PZ-SIC, Perdew and Zunger self-interaction correction.

## References

[b1] Gaillard E. R. & Whitten D. G. Photoinduced electron transfer bond fragmentations. Acc. Chem. Res. 29, 292–297 (1996).

[b2] Newton M. D. Quantum chemical probes of electron-transfer kinetics - the nature of donor-acceptor interactions. Chem. Rev. 91, 767–792 (1991).

[b3] Zewail A. H. Femtochemistry: atomic-scale dynamics of the chemical bond using ultrafast lasers - (Nobel lecture). Angew. Chem. Int. Ed. Engl. 39, 2587–2631 (2000). 10.1002/1521-3773(20000804)39:15<2586::aid-anie2586>3.0.co;2-o 10934390

[b4] Moser C. C., Keske J. M., Warncke K., Farid R. S. & Dutton P. L. Nature of biological electron transfer. Nature 355, 796–802 (1992). 1311417 10.1038/355796a0

[b5] Sanii L. & Schuster G. B. Long-distance charge transport in DNA: Sequence-dependent radical cation injection efficiency. J. Am. Chem. Soc. 122, 11545–11546 (2000).

[b6] Barnett R. N., Cleveland C. L., Joy A., Landman U. & Schuster G. B. Charge migration in DNA: ion-gated transport. Science 294, 567–571 (2001). 11641491 10.1126/science.1062864

[b7] Frischmann P. D., Mahata K. & Wurthner F. Powering the future of molecular artificial photosynthesis with light-harvesting metallosupramolecular dye assemblies. Chem. Soc. Rev. 42, 1847–1870 (2013). 22850767 10.1039/c2cs35223k

[b8] Ge N. H. . Femtosecond dynamics of electron localization at interfaces. Science 279, 202–205 (1998). 9422687 10.1126/science.279.5348.202

[b9] Yeh A. T., Shank C. V. & McCusker J. K. Ultrafast electron localization dynamics following photo-induced charge transfer. Science 289, 935–938 (2000). 10937993 10.1126/science.289.5481.935

[b10] Bakulin A. A. . The role of driving energy and delocalized States for charge separation in organic semiconductors. Science 335, 1340–1344 (2012). 22362882 10.1126/science.1217745

[b11] Falke S. M. . Coherent ultrafast charge transfer in an organic photovoltaic blend. Science 344, 1001–1005 (2014). 24876491 10.1126/science.1249771

[b12] Najafi E., Scarborough T. D., Tang J. & Zewail A. Ultrafast dynamics. Four-dimensional imaging of carrier interface dynamics in p-n junctions. Science 347, 164–167 (2015). 25574020 10.1126/science.aaa0217

[b13] Cohen A. J., Mori-Sanchez P. & Yang W. T. Insights into current limitations of density functional theory. Science 321, 792–794 (2008). 18687952 10.1126/science.1158722

[b14] Jónsson H. Simulation of surface processes. Proc. Natl Acad. Sci. USA 108, 944–949 (2011). 21199939 10.1073/pnas.1006670108 PMC3024682

[b15] Baruah T. & Pederson M. R. Density functional study on a light-harvesting carotenoid-porphyrin-C_60_ molecular triad. J. Chem. Phys. 125, 164706 (2006). 17092119 10.1063/1.2360265

[b16] Perdew J. P. & Zunger A. Self-interaction correction to density-functional approximations for many-electron systems. Phys. Rev. B 23, 5048–5079 (1981).

[b17] Pederson M. R., Heaton R. A. & Lin C. C. Local-density Hartree-Fock theory of electronic states of molecules with self-interaction correction. J. Chem. Phys. 80, 1972–1975 (1984).

[b18] Gudmundsdóttir H., Zhang Y., Weber P. M. & Jónsson H. Self-interaction corrected density functional calculations of molecular Rydberg states. J. Chem. Phys. 139, 194102 (2013). 24320311 10.1063/1.4829539

[b19] Lehtola S. & Jónsson H. Variational, Self-consistent implementation of the Perdew-Zunger self-interaction correction with complex optimal orbitals. J. Chem. Theory Comput. 10, 5324–5337 (2014)) *J. Chem. Theory Comput.* **11**, 5052–5053 (2015). 26574290 10.1021/acs.jctc.5b00806

[b20] Gudmundsdóttir H., Jónsson E. Ö. & Jónsson H. Calculations of Al dopant in α-quartz using a variational implementation of the Perdew-Zunger self-interaction correction. N. J. Phys. 17, 083006 (2015).

[b21] Deb S., Cheng X. & Weber P. M. Structural dynamics and charge transfer in electronically excited N,N'-dimethylpiperazine. J. Phys. Chem. Lett. 4, 2780–2784 (2013).

[b22] Hoffmann R. Interaction of orbitals through space and through bonds. Acc. Chem. Res. 4, 1–9 (1971).

[b23] Brouwer A. M., Langkilde F. W., Bajdor K. & Wilbrandt R. Through-bond interaction in the radical cation of N,N-dimethylpiperazine. Resonance Raman spectroscopy and quantum chemical calculations. Chem. Phys. Lett. 225, 386–390 (1994).

[b24] Brouwer A. M. . Radical cation of N,N-dimethylpiperazine: dramatic structural effects of orbital interactions through bonds. J. Am. Chem. Soc. 120, 3748–3757 (1998).

[b25] Gosselin J. L. & Weber P. M. Rydberg fingerprint spectroscopy: a new spectroscopic tool with local and global structural sensitivity. J. Phys. Chem. A 109, 4899–4904 (2005). 16833836 10.1021/jp0503866

[b26] Kuthirummal N. & Weber P. M. Rydberg states: sensitive probes of molecular structure. Chem. Phys. Lett. 378, 647–653 (2003).

[b27] Cheng X. . Ultrafast structural dynamics in Rydberg excited N,N,N ',N '-tetramethylethylenediamine: conformation dependent electron lone pair interaction and charge delocalization. Chem. Sci. 5, 4394–4403 (2014).

[b28] Cheng X., Zhang Y., Gao Y., Jónsson H. & Weber P. M. Ultrafast structural pathway of charge transfer in N,N,N',N'-tetramethylethylenediamine. J. Phys. Chem. A 119, 2813–2818 (2015). 25714009 10.1021/acs.jpca.5b01797

[b29] Frisch M. J. . *Gaussian 09, Revision* **C.01** (Gaussian, Inc. (2009).

[b30] Frisch M. J. . *Gaussian 09, Revision* **D.01** (Gaussian, Inc. (2009).

[b31] Valiev M. . NWChem: a comprehensive and scalable open-source solution for large scale molecular simulations. Comput. Phys. Commun. 181, 1477–1489 (2010).

[b32] Dunning T. H. Gaussian basis sets for use in correlated molecular calculations. 1. The atoms boron through neon and hydrogen. J. Chem. Phys. 90, 1007–1023 (1989).

[b33] Kendall R. A., Dunning T. H. & Harrison R. J. Electron affinities of the firstrow atoms revisited. Systematic basis sets and wave functions. J. Chem. Phys. 96, 6796–6806 (1992).

[b34] Mortensen J. J., Hansen L. B. & Jacobsen K. W. Real-space grid implementation of the projector augmented wave method. Phys. Rev. B 71, 035109 (2005).

[b35] Enkovaara J. . Electronic structure calculations with GPAW: a real-space implementation of the projector augmented wave method. J. Phys. Condens. Matter 22, 253202 (2010). 21393795 10.1088/0953-8984/22/25/253202

[b36] Valdes A. . Solar hydrogen production with semiconductor metal oxides: new directions in experiment and theory. Phys. Chem. Chem. Phys. 14, 49–70 (2012). 22083224 10.1039/c1cp23212f

[b37] Zhao Y. & Truhlar D. G. Benchmark databases for nonbonded interactions and their use to test density functional theory. J. Chem. Theory Comput. 1, 415–432 (2005). 26641508 10.1021/ct049851d

[b38] Magyar R. J. & Tretiak S. Dependence of spurious charge-transfer excited states on orbital exchange in TDDFT: large molecules and clusters. J. Chem. Theory Comput. 3, 976–987 (2007). 26627417 10.1021/ct600282k

[b39] Zhao Y. & Truhlar D. G. Density functional for spectroscopy: no long-range self-interaction error, good performance for Rydberg and charge-transfer states, and better performance on average than B3LYP for ground states. J. Phys. Chem. A 110, 13126–13130 (2006). 17149824 10.1021/jp066479k

[b40] Zhao Y. & Truhlar D. G. The M06 suite of density functionals for main group thermochemistry, thermochemical kinetics, noncovalent interactions, excited states, and transition elements: two new functionals and systematic testing of four M06-class functionals and 12 other functionals. Theor. Chem. Acc. 120, 215–241 (2008).

[b41] Zhao Y. & Truhlar D. G. Density functionals with broad applicability in chemistry. Acc. Chem. Res. 41, 157–167 (2008). 18186612 10.1021/ar700111a

[b42] Jónsson H., Mills G. & Jacobsen K. W . in Classical and Quantum Dynamics in Condensed Phase Simulations eds Berne B. J., Ciccotti G., Coker D. F. 385–404World Scientific (1998).

[b43] Gudmundsdóttir H., Zhang Y., Weber P. M. & Jónsson H. Self-interaction corrected density functional calculations of Rydberg states of molecular clusters: N,N-dimethylisopropylamine. J. Chem. Phys. 141, 234308 (2014). 25527936 10.1063/1.4902383

[b44] Gavnholt J., Olsen T., Engelund M. & Schiotz J. Delta self-consistent field method to obtain potential energy surfaces of excited molecules on surfaces. Phys. Rev. B 78, 075441 (2008).

