# Supplementary material for: Charge localization in a diamine cation provides a test of energy functionals and self-interaction correction
Source: Nat Commun. 2016 Mar 16;7:11013. doi: 10.1038/ncomms11013 (PMC4799366; doi:10.1038/ncomms11013)
Supplement: Supplementary Information — Supplementary Figures 1-4, Supplementary Tables 1-6, Supplementary Notes 1-7 and Supplementary References [file ncomms11013-s1.pdf]

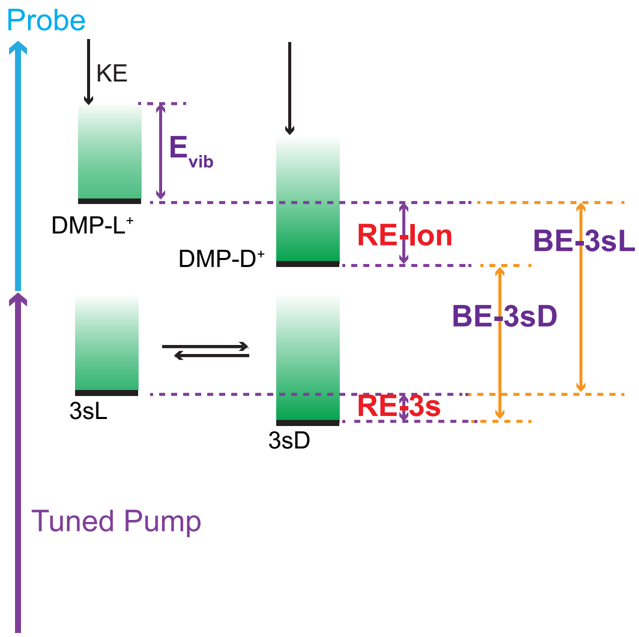

Supplementary Figure 1: Energy of charge-localized, charge-delocalized 3s and ion states. The relative energy (RE) and the binding energy (BE) are labeled in red and purple, resp. The vibrational energy deposited in the molecule is indicated with the green blocks.

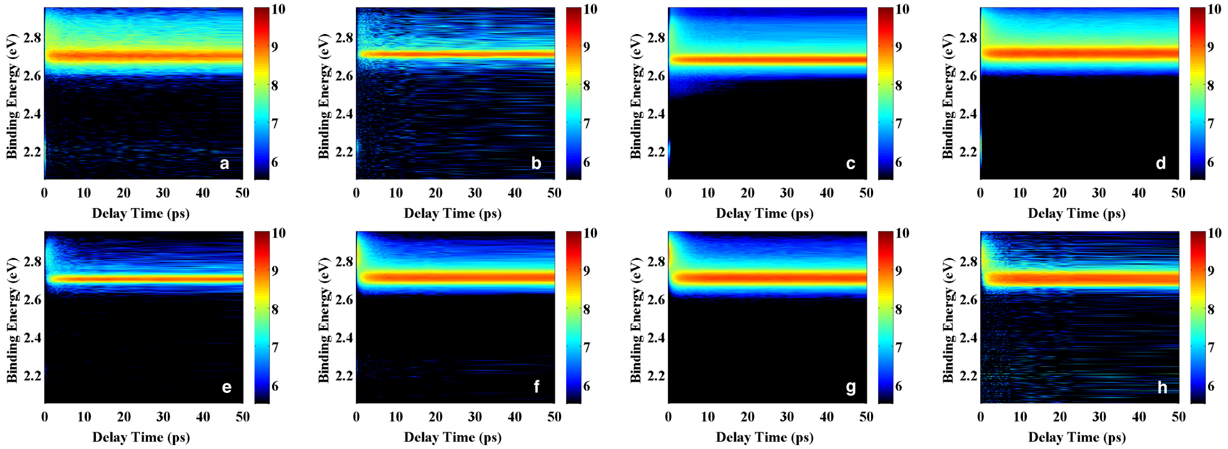

Supplementary Figure 2: The time-dependent photoelectron spectra taken with different pump pulse wavelengths. (**a**) 193.0 nm, (**b**) 200.0 nm, (**c**) 207.0 nm, (**d**) 209.0 nm, (**e**) 220.8 nm, (**f**) 224.9 nm, (**g**) 231.5 nm, and (**h**) 240.8 nm. The colors represent signal intensities on a natural logarithmic scale as given by the color bar (arbitary units).

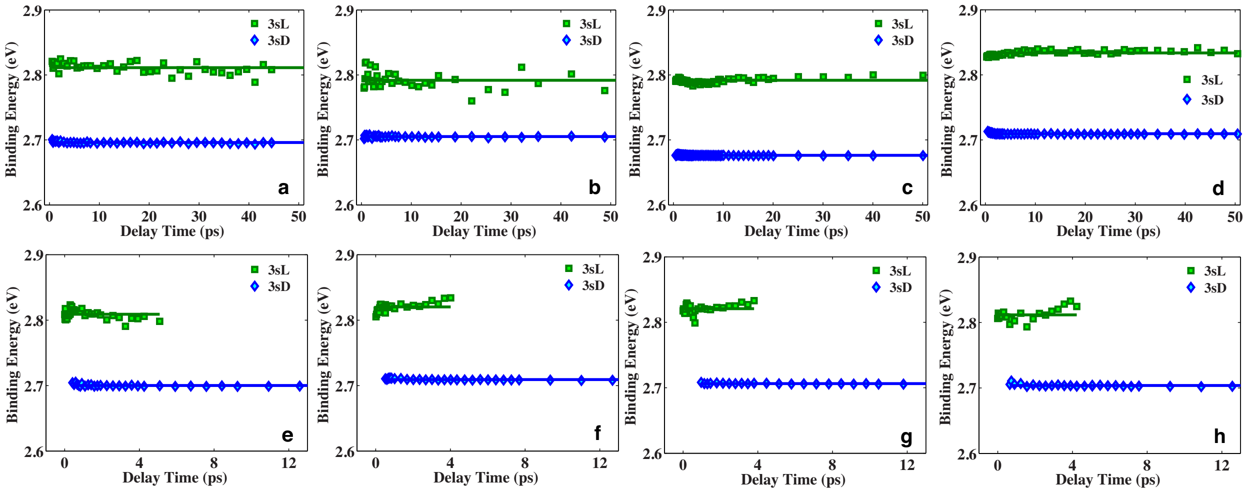

Supplementary Figure 3: The binding energy centers of the deconvoluted 3sL and 3sD peak for different wavelengths. The wavelengths are shown in detail in the caption of Supplementary Figure 2.

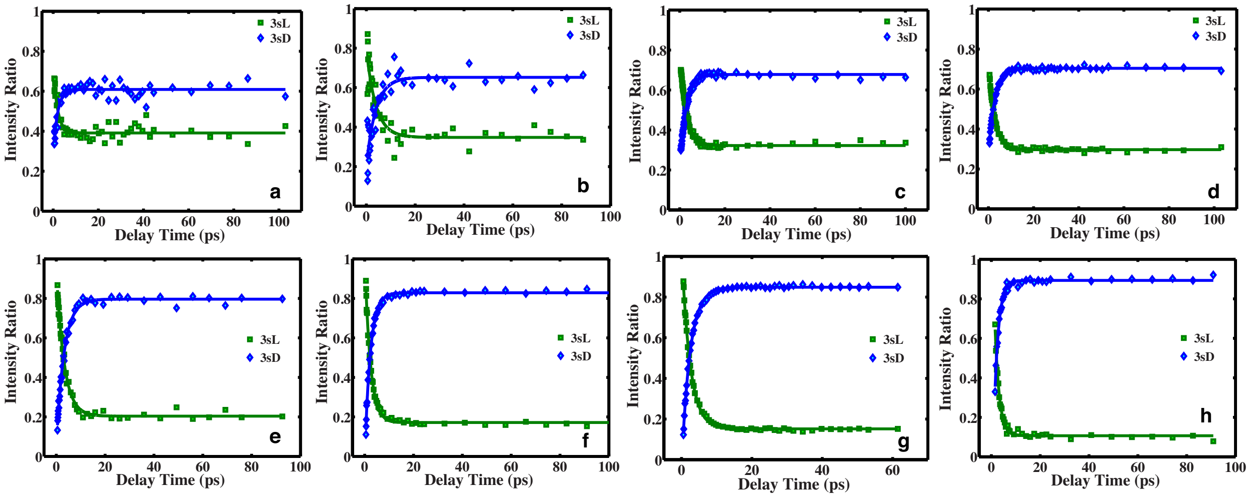

Supplementary Figure 4: Fitted intensity ratio in a suddenly displaced two-component equilibrium system. **(a)** – **(h)** present experiments with the pump wavelengths as given in the caption of Supplementary Figure 2.

Supplementary Table 1: Fitted binding energy centers of the charge-localized and charge-delocalized states. Fitted binding energy centers for the partially overlapping 3s peaks and the average center positions are shown with the uncertainties (3σ) in the parentheses.

λ-pump/nm	3sL/eV	3sD/eV
193.0	2.81	2.70
200.0	2.79	2.71
207.0	2.79	2.68
209.0	2.83	2.71
220.8	2.81	2.70
224.9	2.82	2.71
231.5	2.82	2.71
240.8	2.81	2.70
Average	2.81 (0.04)	2.70 (0.03)

Supplementary Table 2: Fitted intensity ratios and equilibrium constants. Fitted intensity ratios, P(3sD) and P(3sL), for the partially overlapping 3s peaks after equilibrium is reached, and the equilibrium constants, K=P(3sD)/P(3sL), in the experiments with various pump wavelengths are shown in the table. Values in parentheses are the uncertainties (3σ) of the fits.

λ-pump/nm	P(3sD)	P(3sL)	ln(K)
193.0	0.609 (0.016)	0.391 (0.016)	0.443 (0.069)
200.0	0.652 (0.046)	0.348 (0.046)	0.628 (0.201)
207.0	0.678 (0.006)	0.322 (0.006)	0.744 (0.026)
209.0	0.703 (0.004)	0.297 (0.004)	0.864 (0.017)
220.8	0.796 (0.015)	0.204 (0.015)	1.363 (0.090)
224.9	0.829 (0.009)	0.171 (0.009)	1.576 (0.066)
231.5	0.848 (0.005)	0.152 (0.005)	1.723 (0.036)
240.8	0.893 (0.010)	0.107 (0.010)	2.124 (0.100)

Supplementary Table 3: CCSD(T) single point energy of the cation. The atomic structure of DMP-L^+ and DMP-D^+ ions was optimized using HF, MP2, CCSD, conventional DFT with the PBE0 and BHandHLYP functionals, and the PZ-SIC method. (**a**) Using aug-cc-pVDZ basis set. (**b**) The optimizations were repeated for HF, MP2 and BHandHLYP with the cc-pVTZ basis set. The CCSD(T) single point calculation was repeated using the new optimized structures and the cc-pVTZ basis set.

(**a**)

Total Energy of the Ion with aug-cc-pVDZ basis set				
HF	Energy/a.u.	RE/eV	CCSD(T)/a.u.	RE/eV
DMP-D^+	-344.0219305	0	-345.3855158	0
DMP-L^+	-344.0422318	-0.55	-345.3713700	0.39
MP2	Energy/a.u.	RE/eV	CCSD(T)/a.u.	RE/eV
DMP-D^+	-345.2621014	0	-345.3898272	0
DMP-L^+	-345.2330798	0.79	-345.3764118	0.37
BHandHLYP	Energy/a.u.	RE/eV	CCSD(T)/a.u.	RE/eV
DMP-D^+	-346.1036799	0	-345.3857127	0
DMP-L^+	-346.0971438	0.18	-345.3717250	0.38
CCSD	Energy/a.u.	RE/eV	CCSD(T)/a.u.	RE/eV
DMP-D^+	-345.3379692	0	-345.3899978	0
DMP-L^+	-345.3296004	0.23	-345.3768989	0.36
PZ-SIC	Energy/eV	RE/eV	CCSD(T)/a.u.	RE/eV
DMP-D^+	-101.581485	0	-345.3784349	0
DMP-L^+	-101.241740	0.34	-345.3685658	0.27
PBE0	Energy/a.u.	RE/eV	CCSD(T)/a.u.	RE/eV
DMP-D^+	-346.1656619	—	-345.3876414	—

(**b**)

Total Energy of the Ion with cc-pVTZ basis set				
HF	Energy/a.u.	RE/eV	CCSD(T)/a.u.	RE/eV
DMP-D+	-344.1013183	0	-345.6750194	0
DMP-L+	-344.1208376	-0.53	-345.6597813	0.41
MP2	Energy/a.u.	RE/eV	CCSD(T)/a.u.	RE/eV
DMP-D+	-345.5530006	0	-345.6769721	0
DMP-L+	-345.5231728	0.81	-345.6627589	0.39
BHandHLYP	Energy/a.u.	RE/eV	CCSD(T)/a.u.	RE/eV
DMP-D+	-346.1886724	0	-345.6751964	0
DMP-L+	-346.1815073	0.19	-345.6607371	0.39
CCSD[a]	Energy/a.u.	RE/eV	CCSD(T)/a.u.	RE/eV
DMP-D+	—	—	-345.6759645	0
DMP-L+	—	—	-345.6618719	0.38

[a] The CCSD/aug-cc-pVDZ optimized structures were used in the CCSD(T) single point energy calculations here because the CCSD/cc-pVTZ optimizations were found to require too large computational effort.

Supplementary Table 4: Structures and Cartesian coordiantes of DMP. The structures and the Cartesian coordinates of the DMP molecule in the ground state, optimized at (**a**) MP2 and (**b**) CCSD level of theory are shown in the tables.

(**a**) DMP optimized with MP2

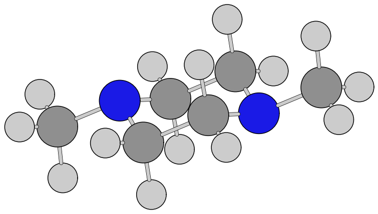

DMP_MP2	Cartesian coordinates (Angstroms)		
Element	x	y	z
N	0.224805	1.423149	0.000000
C	-0.253350	0.719265	1.194143
C	0.253350	-0.719265	1.194143
N	-0.224805	-1.423149	0.000000
C	0.253350	-0.719265	-1.194143
C	-0.253350	0.719265	-1.194143
C	-0.253350	2.804225	0.000000
C	0.253350	-2.804225	0.000000
H	-1.367284	0.713079	1.237584
H	0.124109	1.247669	2.085929
H	-0.124109	-1.247669	2.085929
H	1.367284	-0.713079	1.237584
H	0.124109	1.247669	-2.085929
H	-1.367284	0.713079	-1.237584
H	1.367284	-0.713079	-1.237584
H	-0.124109	-1.247669	-2.085929
H	-1.365241	2.864457	0.000000
H	0.124419	3.324434	-0.893981
H	0.124419	3.324434	0.893981
H	1.365241	-2.864457	0.000000
H	-0.124419	-3.324434	-0.893981
H	-0.124419	-3.324434	0.893981

(**b**) DMP optimized with CCSD

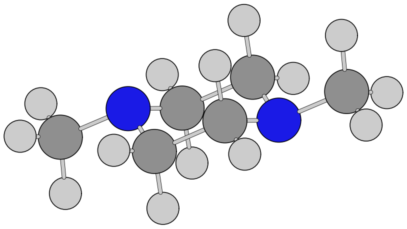

DMP_MP2	Cartesian coordinates (Angstroms)		
Element	x	y	z
N	0.216286	1.427522	0.000000
C	-0.255031	0.721027	1.198452
C	0.255031	-0.721027	1.198452
N	-0.216286	-1.427522	0.000000
C	0.255031	-0.721027	-1.198452
C	-0.255031	0.721027	-1.198452
C	-0.255031	2.812760	0.000000
C	0.255031	-2.812760	0.000000
H	-1.369053	0.713933	1.250817
H	0.126947	1.249128	2.089801
H	-0.126947	-1.249128	2.089801
H	1.369053	-0.713933	1.250817
H	0.126947	1.249128	-2.089801
H	-1.369053	0.713933	-1.250817
H	1.369053	-0.713933	-1.250817
H	-0.126947	-1.249128	-2.089801
H	-1.367585	2.882962	0.000000
H	0.126706	3.332788	-0.894771
H	0.126706	3.332788	0.894771
H	1.367585	-2.882962	0.000000
H	-0.126706	-3.332788	-0.894771
H	-0.126706	-3.332788	0.894771

Supplementary Table 5: Structures and Cartesian coordiantes of DMP$^+$. The structures and the Cartesian coordinates of the charge-localized DMP-L$^+$ ion and charge-delocalized DMP-D$^+$ ion optimized by HF, MP2, CCSD, PZ-SIC and conventional DFT with the BHandHLYP and PBE0 functionals are shown in the tables (**a**) – (**k**).

(**a**) DMP-L$^+$ optimized with HF

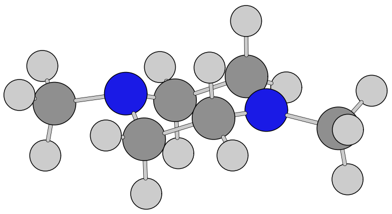

With aug-cc-pVDZ basis set:

DMP-L$^+$_HF	Cartesian coordinates (Angstroms)		
Element	x	y	z
N	-1.385080	-0.001648	-0.255152
C	-0.730374	1.186625	0.244426
C	0.696931	1.264790	-0.289458
N	1.415580	0.005813	-0.067207
C	0.702704	-1.256142	-0.291870
C	-0.724315	-1.186687	0.243502
C	-2.816421	-0.005195	0.020800
C	2.840009	-0.002990	0.235936
H	-0.715595	1.226800	1.341853
H	-1.253190	2.072372	-0.106895
H	1.264196	2.064301	0.176096
H	0.691743	1.411269	-1.371097
H	-1.242796	-2.075007	-0.107841
H	-0.708645	-1.227624	1.340968
H	0.697194	-1.399983	-1.373746
H	1.275289	-2.053810	0.170939
H	-3.272845	0.873667	-0.427407
H	-3.036838	-0.005809	1.094730
H	-3.268424	-0.886311	-0.427472
H	3.308215	-0.839154	-0.274375
H	2.951772	-0.128230	1.314152
H	3.285217	0.935962	-0.073409

With cc-pVTZ basis set:

DMP-L+_HF	Cartesian coordinates (Angstroms)		
Element	x	y	z
N	0.720455	1.202576	0.000000
C	0.027458	0.763970	1.184326
C	0.027458	-0.757247	1.257498
N	-0.433993	-1.345857	0.000000
C	0.027458	-0.757247	-1.257498
C	0.027458	0.763970	-1.184326
C	0.985145	2.631999	0.000000
C	-1.233903	-2.558856	0.000000
H	-1.000022	1.132170	1.226912
H	0.536091	1.127128	2.065478
H	-0.600372	-1.127248	2.052949
H	1.037208	-1.127966	1.400476
H	0.536091	1.127128	-2.065478
H	-1.000022	1.132170	-1.226912
H	1.037208	-1.127966	-1.400476
H	-0.600372	-1.127248	-2.052949
H	1.564213	2.891374	0.874405
H	0.071880	3.226280	0.000000
H	1.564213	2.891374	-0.874405
H	-1.019392	-3.132612	-0.887410
H	-2.279009	-2.268553	0.000000
H	-1.019392	-3.132612	0.887410

(**b**) DMP-D$^+$ optimized with HF

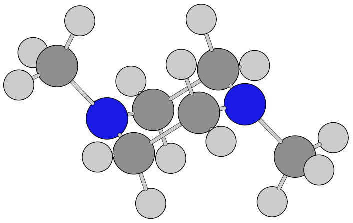

With aug-cc-pVDZ basis set:

DMP-D$^+$_HF	Cartesian coordinates (Angstroms)		
Element	x	y	z
N	0.232020	1.385041	0.000000
C	-0.232020	0.774264	1.186092
C	0.232020	-0.774264	1.186092
N	-0.232020	-1.385041	0.000000
C	0.232020	-0.774264	-1.186092
C	-0.232020	0.774264	-1.186092
C	1.558369	1.986895	0.000000
C	-1.558369	-1.986895	0.000000
H	-1.317991	0.785464	1.230631
H	0.177979	1.252343	2.069046
H	-0.177979	-1.252343	2.069046
H	1.317991	-0.785464	1.230631
H	0.177979	1.252343	-2.069046
H	-1.317991	0.785464	-1.230631
H	1.317991	-0.785464	-1.230631
H	-0.177979	-1.252343	-2.069046
H	1.670110	2.607465	0.883069
H	1.670110	2.607465	-0.883069
H	2.344777	1.225810	0.000000
H	-1.670110	-2.607465	0.883069
H	-1.670110	-2.607465	-0.883069
H	-2.344777	-1.225810	0.000000

With cc-pVTZ basis set:

DMP-D^{+}_HF	Cartesian coordinates (Angstroms)		
Element	x	y	z
N	0.231763	1.379698	0.000000
C	-0.231763	0.774878	1.182646
C	0.231763	-0.774878	1.182646
N	-0.231763	-1.379698	0.000000
C	0.231763	-0.774878	-1.182646
C	-0.231763	0.774878	-1.182646
C	1.545858	1.999230	0.000000
C	-1.545858	-1.999230	0.000000
H	-1.311332	0.782824	1.2271^{49}
H	0.173883	1.250803	2.060452
H	-0.173883	-1.250803	2.060452
H	1.311332	-0.782824	1.227149
H	0.173883	1.250803	-2.060452
H	-1.311332	0.782824	-1.227149
H	1.311332	-0.782824	-1.227149
H	-0.173883	-1.250803	-2.060452
H	1.649799	2.617597	0.877388
H	1.649799	2.617597	-0.877388
H	2.338780	1.254558	0.000000
H	-1.649799	-2.617597	0.877388
H	-1.649799	-2.617597	-0.877388
H	-2.344777	-1.225810	0.000000

(**c**) DMP-L$^+$ optimized with MP2

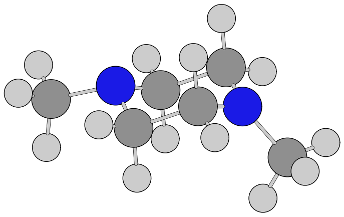

With aug-cc-pVDZ basis set:

DMP-L$^+$_MP2	Cartesian coordinates (Angstroms)		
Element	x	y	z
N	0.866786	1.070241	0.000000
C	0.161517	0.628293	1.193909
C	0.161517	-0.925779	1.228311
N	-0.461814	-1.379188	0.000000
C	0.161517	-0.925779	-1.228311
C	0.161517	0.628293	-1.193909
C	1.045978	2.533795	0.000000
C	-1.831870	-1.850938	0.000000
H	-0.888392	0.995942	1.241474
H	0.691824	0.978821	2.092356
H	-0.393853	-1.317206	2.090169
H	1.205210	-1.276537	1.236009
H	0.691824	0.978821	-2.092356
H	-0.888392	0.995942	-1.241474
H	1.205210	-1.276537	-1.236009
H	-0.393853	-1.317206	-2.090169
H	1.618016	2.823900	0.891844
H	0.077342	3.073331	0.000000
H	1.618016	2.823900	-0.891844
H	-2.018116	-2.440042	-0.907088
H	-2.502580	-0.967760	0.000000
H	-2.018116	-2.440042	0.907088

With cc-pVTZ basis set:

DMP-L^+_MP2	Cartesian coordinates (Angstroms)		
Element	x	y	z
N	0.852383	1.066103	0.000000
C	0.156232	0.628409	1.186446
C	0.156232	-0.916254	1.219872
N	-0.461173	-1.365961	0.000000
C	0.156232	-0.916254	-1.219872
C	0.156232	0.628409	-1.186446
C	1.054143	2.515882	0.000000
C	-1.814870	-1.854668	0.000000
H	-0.882840	0.990482	1.238101
H	0.681790	0.974867	2.074595
H	-0.390151	-1.306330	2.072928
H	1.190635	-1.258392	1.227646
H	0.681790	0.974867	-2.074595
H	-0.882840	0.990482	-1.238101
H	1.190635	-1.258392	-1.227646
H	-0.390151	-1.306330	-2.072928
H	1.624544	2.795912	0.881626
H	0.106297	3.066906	0.000000
H	1.624544	2.795912	-0.881626
H	-1.992706	-2.440826	-0.896309
H	-2.492519	-0.992468	0.000000
H	-1.992706	-2.440826	0.896309

(**d**) DMP-D^+ optimized with MP2

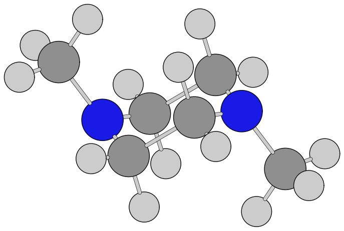

With aug-cc-pVDZ basis set:

DMP-D^+_MP2	Cartesian coordinates (Angstroms)		
Element	x	y	z
N	0.237938	1.393907	0.000000
C	-0.237938	0.767756	1.196198
C	0.237938	-0.767756	1.196198
N	-0.237938	-1.393907	0.000000
C	0.237938	-0.767756	-1.196198
C	-0.237938	0.767756	-1.196198
C	1.616457	1.886829	0.000000
C	-1.616457	-1.886829	0.000000
H	-1.339277	0.775630	1.219701
H	0.168116	1.263191	2.089621
H	-0.168116	-1.263191	2.089621
H	1.339277	-0.775630	1.219701
H	0.168116	1.263191	-2.089621
H	-1.339277	0.775630	-1.219701
H	1.339277	-0.775630	-1.219701
H	-0.168116	-1.263191	-2.089621
H	1.777888	2.500985	0.895881
H	1.777888	2.500985	-0.895881
H	2.346718	1.053251	0.000000
H	-1.777888	-2.500985	0.895881
H	-1.777888	-2.500985	-0.895881
H	-2.346718	-1.053251	0.000000

With cc-pVTZ basis set:

DMP-D^+_MP2	Cartesian coordinates (Angstroms)		
Element	x	y	z
N	0.236430	1.381898	0.000000
C	-0.236430	0.763027	1.188049
C	0.236430	-0.763027	1.188049
N	-0.236430	-1.381898	0.000000
C	0.236430	-0.763027	-1.188049
C	-0.236430	0.763027	-1.188049
C	1.597879	1.890502	0.000000
C	-1.597879	-1.890502	0.000000
H	-1.326184	0.768195	1.210767
H	0.162028	1.255181	2.071867
H	-0.162028	-1.255181	2.071867
H	1.326184	-0.768195	1.210767
H	0.162028	1.255181	-2.071867
H	-1.326184	0.768195	-1.210767
H	1.326184	-0.768195	-1.210767
H	-0.162028	-1.255181	-2.071867
H	1.751775	2.500408	0.885514
H	1.751775	2.500408	-0.885514
H	2.332161	1.076707	0.000000
H	-1.751775	-2.500408	0.885514
H	-1.751775	-2.500408	-0.885514
H	-2.332161	-1.076707	0.000000

(**e**) DMP-L$^+$ optimized with CCSD

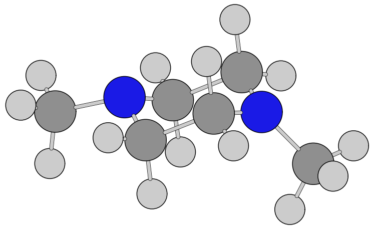

DMP-L$^+$_CCSD	Cartesian coordinates (Angstroms)		
Element	x	y	z
N	0.849247	1.078575	0.000000
C	0.151752	0.638771	1.199958
C	0.151752	-0.919023	1.237097
N	-0.467129	-1.382654	0.000000
C	0.151752	-0.919023	-1.237097
C	0.151752	0.638771	-1.199958
C	1.098892	2.531972	0.000000
C	-1.838022	-1.878937	0.000000
H	-0.900181	0.999944	1.254346
H	0.686725	0.995365	2.094089
H	-0.412001	-1.309002	2.095690
H	1.193763	-1.276687	1.256159
H	0.686725	0.995365	-2.094089
H	-0.900181	0.999944	-1.254346
H	1.193763	-1.276687	-1.256159
H	-0.412001	-1.309002	-2.095690
H	1.685512	2.795432	0.893312
H	0.157822	3.120325	0.000000
H	1.685512	2.795432	-0.893312
H	-2.011577	-2.475028	-0.906848
H	-2.524393	-1.007006	0.000000
H	-2.011577	-2.475028	0.906848

(**f**) DMP-D^+ optimized with CCSD

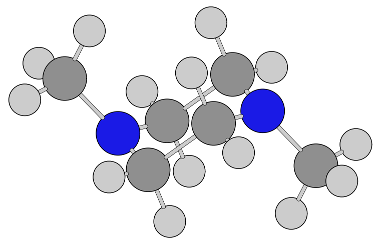

DMP-D^+_CCSD	Cartesian coordinates (Angstroms)		
Element	x	y	z
N	0.236838	1.394816	0.000000
C	-0.236838	0.772763	1.197551
C	0.236838	-0.772763	1.197551
N	-0.236838	-1.394816	0.000000
C	0.236838	-0.772763	-1.197551
C	-0.236838	0.772763	-1.197551
C	1.606702	1.920596	0.000000
C	-1.606702	-1.920596	0.000000
H	-1.338427	0.782281	1.228704
H	0.173368	1.264996	2.091280
H	-0.173368	-1.264996	2.091280
H	1.338427	-0.782281	1.228704
H	0.173368	1.264996	-2.091280
H	-1.338427	0.782281	-1.228704
H	1.338427	-0.782281	-1.228704
H	-0.173368	-1.264996	-2.091280
H	1.754898	2.539344	0.896632
H	1.754898	2.539344	-0.896632
H	2.355049	1.101783	0.000000
H	-1.754898	-2.539344	0.896632
H	-1.754898	-2.539344	-0.896632
H	-2.355049	-1.101783	0.000000

(g) DMP-L$^+$ optimized with PZ-SIC

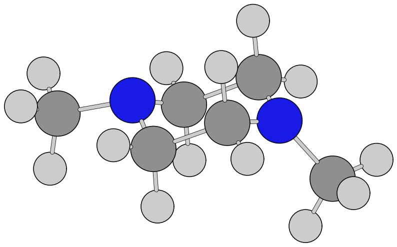

DMP-L+_PZ-SIC	Cartesian coordinates (Angstroms)		
Element	x	y	z
N	11.251018	10.730470	10.000003
C	10.555428	10.309069	11.183184
C	10.557634	8.780613	11.218623
N	9.964760	8.300654	10.000000
C	10.557633	8.780612	8.781384
C	10.555436	10.309068	8.816825
C	11.491757	12.163079	10.000002
C	8.606998	7.834732	10.000000
H	9.530348	10.681586	11.238744
H	11.091973	10.654517	12.057235
H	10.005578	8.394718	12.062161
H	11.582093	8.429266	11.234021
H	11.091976	10.654521	7.942768
H	9.530340	10.681592	8.761261
H	11.582091	8.429269	8.765981
H	10.005577	8.394721	7.937839
H	12.067243	12.417212	10.879430
H	10.567856	12.742158	10.000002
H	12.067242	12.417208	9.120575
H	8.441301	7.246433	9.109100
H	7.943422	8.697032	10.000001
H	8.441301	7.246433	10.890902

(**h**) DMP-D^{+} optimized with PZ-SIC

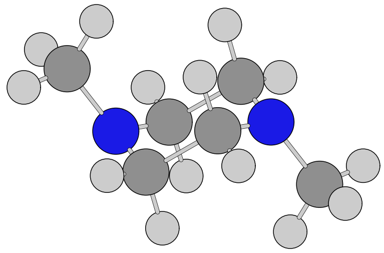

DMP-D+_PZ-SIC	Cartesian coordinates (Angstroms)		
Element	x	y	z
N	10.232760	11.399145	9.999424
C	9.766531	10.755139	11.152707
C	9.770635	10.752757	8.843099
C	11.582707	11.901388	9.997519
N	9.767240	8.600855	9.999424
C	10.233469	9.244861	11.152707
C	10.229365	9.247243	8.843099
C	8.417293	8.098612	9.997519
H	10.179504	11.191883	12.050747
H	8.682917	10.768495	11.212669
H	8.686967	10.776744	8.788483
H	10.179130	11.195975	7.945922
H	11.728937	12.510093	10.878747
H	11.730569	12.513552	9.118971
H	12.336887	11.109173	9.997642
H	9.820496	8.808117	12.050747
H	11.317083	9.231505	11.212669
H	11.313033	9.223256	8.788483
H	9.820870	8.804025	7.945922
H	8.271063	7.489907	10.878747
H	8.269431	7.486448	9.118971
H	7.663113	8.890827	9.997642

(**i**) DMP-L$^+$ optimized with BHandHLYP

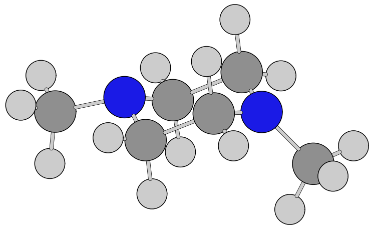

With aug-cc-pVDZ basis set:

DMP-L$^+$_BHandHLYP	Cartesian coordinates (Angstroms)		
Element	x	y	z
N	-1.378932	-0.003352	-0.226901
C	-0.705445	1.187327	0.222762
C	0.700124	1.248790	-0.380457
N	1.410727	0.013527	-0.128835
C	0.713221	-1.228795	-0.388546
C	-0.690703	-1.187437	0.218188
C	-2.794842	-0.012378	0.105594
C	2.757891	-0.005867	0.378014
H	-0.629913	1.247429	1.319155
H	-1.245791	2.068554	-0.118935
H	1.280830	2.076787	0.016026
H	0.618690	1.350018	-1.467284
H	-1.221707	-2.073941	-0.124470
H	-0.612504	-1.249663	1.314463
H	0.632766	-1.324689	-1.475509
H	1.306062	-2.051484	0.002621
H	-3.272092	0.866545	-0.323696
H	-2.973101	-0.015002	1.188882
H	-3.261381	-0.895772	-0.326273
H	3.335898	-0.751465	-0.168224
H	2.728250	-0.305096	1.431918
H	3.209944	0.976712	0.288157

With cc-pVTZ basis set:

DMP-L^+_BHandHLYP	Cartesian coordinates (Angstroms)		
Element	x	y	z
N	-1.367661	-0.000001	-0.201805
C	-0.677568	1.185977	0.214382
C	0.707003	1.231585	-0.437485
N	1.410658	0.000004	-0.173449
C	0.707008	-1.231579	-0.437487
C	-0.677564	-1.185977	0.214380
C	-2.777959	-0.000004	0.139852
C	2.701427	-0.000002	0.455791
H	-0.561544	1.255017	1.299836
H	-1.220385	2.062352	-0.111982
H	1.297596	2.060682	-0.080029
H	0.585544	1.309149	-1.513794
H	-1.220377	-2.062354	-0.111985
H	-0.561539	-1.255019	1.299834
H	0.585550	-1.309142	-1.513797
H	1.297604	-2.060674	-0.080033
H	-3.250764	0.874985	-0.283552
H	-2.948882	-0.000005	1.216461
H	-3.250761	-0.874994	-0.283554
H	3.246749	-0.889544	0.178152
H	2.559447	-0.000090	1.536347
H	3.246701	0.889609	0.178282

(**j**) DMP-D^+ optimized with BHandHLYP

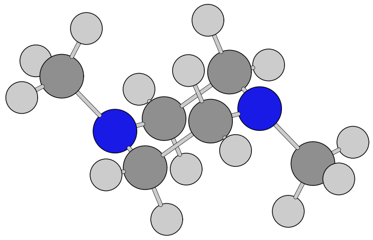

With aug-cc-pVDZ basis set:

DMP-D^+_BHandHLYP	Cartesian coordinates (Angstroms)		
Element	x	y	z
N	0.230328	1.382217	0.000000
C	-0.230328	0.766153	1.187128
C	0.230328	-0.766153	1.187128
N	-0.230328	-1.382217	0.000000
C	0.230328	-0.766153	-1.187128
C	-0.230328	0.766153	-1.187128
C	1.549231	1.981621	0.000000
C	-1.549231	-1.981621	0.000000
H	-1.319094	0.779115	1.230077
H	0.178186	1.251643	2.069271
H	-0.178186	-1.251643	2.069271
H	1.319094	-0.779115	1.230077
H	0.178186	1.251643	-2.069271
H	-1.319094	0.779115	-1.230077
H	1.319094	-0.779115	-1.230077
H	-0.178186	-1.251643	-2.069271
H	1.664450	2.602615	0.884738
H	1.664450	2.602615	-0.884738
H	2.339925	1.220932	0.000000
H	-1.664450	-2.602615	0.884738
H	-1.664450	-2.602615	-0.884738
H	-2.339925	-1.220932	0.000000

With cc-pVTZ basis set:

DMP-D$^+$_BHandHLYP	Cartesian coordinates (Angstroms)		
Element	x	y	z
N	0.229722	1.377307	0.000000
C	-0.229722	0.766216	1.183908
C	0.229722	-0.766216	1.183908
N	-0.229722	-1.377307	0.000000
C	0.229722	-0.766216	-1.183908
C	-0.229722	0.766216	-1.183908
C	1.537075	1.993936	0.000000
C	-1.537075	-1.993936	0.000000
H	-1.311278	0.775771	1.227445
H	0.174256	1.249020	2.060365
H	-0.174256	-1.249020	2.060365
H	1.311278	-0.775771	1.227445
H	0.174256	1.249020	-2.060365
H	-1.311278	0.775771	-1.227445
H	1.311278	-0.775771	-1.227445
H	-0.174256	-1.249020	-2.060365
H	1.644766	2.612345	0.878370
H	1.644766	2.612345	-0.878370
H	2.333240	1.249904	0.000000
H	-1.644766	-2.612345	0.878370
H	-1.644766	-2.612345	-0.878370
H	-2.333240	-1.249904	0.000000

(**k**) DMP-D^+ optimized with PBE0

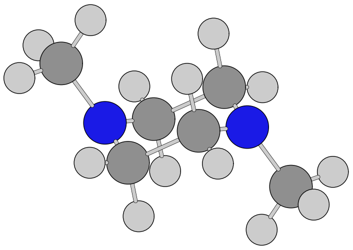

DMP-D^+_PBE0	Cartesian coordinates (Angstroms)		
Element	x	y	z
N	0.230222	1.387213	0.000000
C	-0.230222	0.762752	1.189371
C	0.230222	-0.762752	1.189371
N	-0.230222	-1.387213	0.000000
C	0.230222	-0.762752	-1.189371
C	-0.230222	0.762752	-1.189371
C	1.549298	1.983941	0.000000
C	-1.549298	-1.983941	0.000000
H	-1.327915	0.777697	1.229801
H	0.177082	1.255334	2.078488
H	-0.177082	-1.255334	2.078488
H	1.327915	-0.777697	1.229801
H	0.177082	1.255334	-2.078488
H	-1.327915	0.777697	-1.229801
H	1.327915	-0.777697	-1.229801
H	-0.177082	-1.255334	-2.078488
H	1.668292	2.609201	0.891053
H	1.668292	2.609201	-0.891053
H	2.347701	1.218838	0.000000
H	-1.668292	-2.609201	0.891053
H	-1.668292	-2.609201	-0.891053
H	-2.347701	-1.218838	0.000000

Supplementary Table 6: Structure and Cartesian coordiante of the saddle point of DMP^+. The structures and the Cartesian coordinates of the saddle point (SP) configuration of DMP^+ ion is shown in the table.

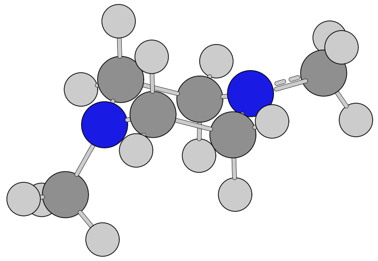

DMP^+-SP_PZ-SIC	Cartesian coordinates (Angstroms)		
Element	x	y	z
N	10.822400	10.986898	9.928614
C	10.300678	10.522941	11.165749
C	10.442612	8.989740	11.232509
N	9.854461	8.445801	10.058409
C	10.450925	8.859350	8.850395
C	10.309503	10.392219	8.740073
C	11.403340	12.229243	9.832602
C	8.487996	7.998958	10.043213
H	9.249742	10.771417	11.251062
H	10.837810	10.975500	11.983548
H	9.950841	8.618597	12.111972
H	11.488823	8.725714	11.263255
H	10.853656	10.749777	7.888028
H	9.259285	10.629582	8.606334
H	11.496744	8.596640	8.848047
H	9.949736	8.387843	8.023573
H	11.845182	12.535920	10.755680
H	10.691714	12.930646	9.579043
H	12.098932	12.174525	9.070330
H	8.409564	7.069354	9.586286
H	7.893749	8.674797	9.499888
H	8.104102	7.936510	11.010139

Supplementary Note 1: The Self-interaction Error and Implementation of PZ-SIC

The difficulty currently available DFT functionals have in describing localized states can be understood by considering the self-interaction error. Kohn-Sham DFT strives to obtain the energy of the electronic system as a functional of only the total electron density associated with each spin component, $\rho_\uparrow(\mathbf{r})$ and $\rho_\downarrow(\mathbf{r})$

$$E^{\mathrm{KS}}[\rho_\uparrow,\rho_\downarrow]=T_s[\rho_\uparrow,\rho_\downarrow]+V_{\mathrm{ext}}[\rho]+E_{\mathrm{C}}^{\mathrm{KS}}[\rho]+E_{\mathrm{xc}}[\rho_\uparrow,\rho_\downarrow] \tag{1}$$

where $\rho=\rho_\uparrow+\rho_\downarrow$. Here, T_s is the kinetic energy of an independent electron reference system that has the same electron density as the real system, V_{ext} is the external potential representing the interaction of the electrons with the nuclei, $E_{\mathrm{C}}^{\mathrm{KS}}$ is an estimate of the Coulomb repulsion between the electrons, and E_{xc} is the exchange and correlation energy. While a set of orthonormal orbitals is introduced in order to obtain a better estimate of the kinetic energy, they are not used to estimate other contributions to the total energy. The electron-electron Coulomb repulsion is estimated from the total electron density as

$$E_{\mathrm{C}}^{\mathrm{KS}}[\rho]=\frac{1}{2}\iint d^3\mathbf{r}d^3\mathbf{r}'\frac{\rho(\mathbf{r})\rho(\mathbf{r}')}{|\mathbf{r}-\mathbf{r}'|} \tag{2}$$

an expression that clearly represents only a spurious self-interaction when the system contains a single electron. For many electron systems, one can rewrite this expression in terms of orbital densities, where $\rho(\mathbf{r})=\sum_{m\sigma}\rho_{m\sigma}(\mathbf{r})$ as

$$E_{\mathrm{C}}^{\mathrm{KS}}=\frac{1}{2}\iint d^3\mathbf{r}d^3\mathbf{r}'\sum_{m\sigma}\sum_{m'\sigma'}\frac{\rho_{m\sigma}(\mathbf{r})\rho_{m'\sigma'}(\mathbf{r}')}{|\mathbf{r}-\mathbf{r}'|} \tag{3}$$

where the double sum includes the diagonal, self-interaction terms

$$E_{\mathrm{C}}^{\mathrm{SI}}=\frac{1}{2}\iint d^3\mathbf{r}d^3\mathbf{r}'\sum_{m\sigma}\frac{\rho_{m\sigma}(\mathbf{r})\rho_{m\sigma}(\mathbf{r}')}{|\mathbf{r}-\mathbf{r}'|} \tag{4}$$

representing Coulomb interaction of the orbital densities with themselves. This is the Coulomb part of the self-interaction error in KS-DFT. The term $E_{\mathrm{C}}^{\mathrm{KS}}$ is often referred to as the 'Hartree energy', even though Hartree in his early calculations of the electronic structure of atoms did not include the diagonal, self-interaction terms[45-47].

One of the tasks of the $E_{xc}[\rho_\uparrow,\rho_\downarrow]$ term in the KS-DFT functional is to cancel out these self-interaction terms. Such a cancellation does occur in Hartree-Fock theory where exchange is calculated exactly. However, functionals developed for KS-DFT typically only obtain partial cancellation and, therefore, include a self-interaction error which is the source of several inaccuracies of such calculations, in particular the destabilization of localized electronic states. The more localized an orbital is, the larger the repulsive self-interaction Coulomb energy is and, thereby, an artificial tendency for delocalization. The activation energy for chemical reactions is also typically underestimated in KS-DFT calculations employing semi-local functionals because the transition state is typially less localized and has a smaller repulsive self-interaction error than stable states.[48]

Perdew and Zunger proposed a procedure where a KS-DFT functional is corrected by explicitly subtracting an orbital based estimate of the self-interaction[16]. The corrected energy functional, E^{SIC}, is

$$E^{\mathrm{SIC}}[\{\rho_{m\sigma}\}] = E^{\mathrm{KS}}[\rho_\uparrow,\rho_\downarrow] - \sum_{m\sigma}(E_{\mathrm{C}}^{\mathrm{KS}}[\rho_{m\sigma}] + E_{\mathrm{xc}}[\rho_{m\sigma},0]) \qquad (5)$$

where the spin density for spin component σ is $\rho_\sigma = \sum_m \rho_{m\sigma}$. This correction procedure cancels out the diagonal terms in $E_{\mathrm{C}}^{\mathrm{KS}}$ and gives a self-interaction free functional for one-electron systems where E_{xc} vanishes, but for many-electron systems this orbital based estimate of the self-interaction in $E_{\mathrm{xc}}[\rho_\uparrow,\rho_\downarrow]$ is only approximate. Unlike a KS-DFT functional, the total energy given by E^{SIC} depends on the orbital densities, $\rho_{m\sigma}$. As a result, the variational minimization is more challenging[19, 49]. The present calculations make use of a variational, self-consistent implementation of PZ-SIC applied equally to all occupied orbitals. Recently, it has been realized that it is important not to restrict the orbitals to real valued functions[48, 50]. While variational, self-consistent calculations of E^{SIC} using complex optimal orbitals have been used to study molecules and even clusters of molecules[18, 43, 48], the present work represents the first application where the energy difference between a localized state and a delocalized state of the same molecule is studied.

Supplementary Note 2: Experimental and Computational Methods

The vacuum and spectrometer setup has been described before[51, 52]. The data taken with 207.0 nm pump pulses was previously reported[21]. The data with other wavelengths were taken with a modified experimental setup. A two-stage amplifier (a regenerative amplifier followed by a single pass amplifier, Coherent Legend Elite Duo) produced the fundamental laser beam at 808 nm with a 5 kHz repetition rate. 90% of the fundamental beam was sent to the optical parametric amplifier (OPA, Coherent Opera SOLO) to generate the wavelength tunable pump pulses, while 10% was upconverted by a BBO crystal to the second harmonic (404.0 nm) to be used as probe pulses. The pump and probe laser beams were perpendicularly focused with a 500 mm concave mirror onto the molecular beam, which was generated by entraining DMP at 0 °C in a stream of 1.1 bar of helium carrier gas and expanding through a 100 μm nozzle and a 150 μm skimmer. The peak intensities at the focus of the pump and probe pulses were estimated on the order of 10^{10} W·cm^{-2} and 10^{11} W·cm^{-2}, respectively. Photoelectrons were recorded using a linear time-of-flight spectrometer. The binding energies were determined by subtracting the electron kinetic energies from the probe photon energy. The delay time was adjusted by changing the difference of the optical path length between the pump and probe pulses using a linear delay stage. The time zero was determined by monitoring the two-color mass signal from the OPA pulse excitation and second harmonic ionization of DMP. The cross-correlation times between the pump and probe pulses were measured to be 100 – 150 fs at fwhm. DMP (98%) was purchased from Sigma-Aldrich and used without further purification.

The functionals tested in the report include: B3LYP, B3P86, B3PW91, B1B95, mPW1PW91, mPW1LYP, mPW1PBE, mPW3PBE, B98, B971, B972, PBE1PBE, B1LYP, O3LYP, BHandH, BHandHLYP, BMK, M06, M06HF, M062X, tHCTHhyb, APFD, APF, SOGGA11X, PBEh1PBE, TPSSh, X3LYP, TPSSTPSS, PBEPBE, BB1K HSEH1PBE, wB97XD, wB97, wB97X, LC-wPBE, O3LYP and CAM-B3LYP. The DFT calculations with the APFD, APF and SOGGA11X functionals were implemented with Gaussian 09, Revision D.01[30]; DFT calculations with the B1B95, O3LYP and BB1K functionals were carried out using the NWChem software[31]; DFT calculation with all other functionals were carried out using Gaussian 09, Revision C.01[29]. Key word "Int=UltraFine" were used in all DFT calculations in Gaussian to ensure sufficiently fine integration grids. For M06HF, "Int=SuperFineGrid" was also tested for comparison. The results show the same trend regardless of which integration grids used. To

obtain the charge-localized DMP-L$^+$ structure, the symmetry of the initial input was manually broken by making one of the nitrogen atoms semi-planar and rotating both methyl groups out of the equilibrium position of the ground state structure. All of them except one failed to converge to a stable localized state, even starting from an asymmetric initial geometry. The only exception was the BHandHLYP functional, which has been found to have good results on CT interaction problems while generally giving poor results of other properties such as energy (and therefore not used commonly).

The CCSD structure optimizations, CCSD(T) single point energy calculations and the EOM-CCSD excitation energy calculations were all carried out with the Gaussian 09 program[29]. The MP2 optimized DMP-L$^+$ and DMP-D$^+$ ion structures were used in the binding energy calculations. Assuming CCSD(T) gives a better total energy than CCSD, the binding energies of EOM-CCSD were calculated by subtracting the EOM-CCSD vertical excitation energy from the CCSD(T) vertical ionization potential.

The PZ-SIC calculations, including the structural optimization and calculation of the Rydberg states and corresponding binding energies[16], were carried out using the GPAW program[34-36] using a uniform, real space grid representation of the wavefunctions. The PZ-SIC was applied to the PBE functional[53] in the structural optimizations and calculations of the relative energy of the DMP-L$^+$ and DMP-D$^+$ ion states, using a cubic simulation cell of 20-Å side length and 0.13-Å grid spacing. The Rydberg state binding energy calculations were carried out with PZ-SIC applied to the LDA functional[54] and a cubic simulation cell of 25-Å side length and 0.15-Å grid spacing. For calculating the binding energies, the Rydberg orbitals were first obtained using PZ-SIC for the ground state. The total energy of the Rydberg excited state was then calculated using PZ-SIC and the Delta Self-Consistent Field method[44] where one electron was removed from the highest occupied molecular orbital (HOMO) and placed in the desired Rydberg orbital. The binding energy of the Rydberg state was calculated by subtracting the total energy of the Rydberg excited state from that of the ion.

The minimum energy paths between the localized and delocalized state of the cation, shown in Fig. 3, were calculated using the climbing-image nudged elastic band method[55] with tangent estimate and interpolation method presented in ref. 56. The initial path was generated using the IDPP method[57] and rotation was removed using a quaternion based method[58] as

implemented in the atomic simulation environment (ASE). The PZ-SIC calculations made use of the PBE functional and were carried out using the GPAW software. The M06-HF calculations were carried out using the NWCHEM software.

Supplementary Note 3: The Energy Diagram

To further compare the relative energy shown in Supplementary Fig. 1b, the zero-point energy correction to the vibrational ground state was estimated from harmonic vibrational frequencies calculated using MP2 for the ground state of the neutral and cationic states. The difference in zero point energy between the two states turns out to be 0.07 eV and the corrected relative energy obtained from the CCSD(T) calculations then becomes 0.37-0.07 = 0.30 eV. The PZ-SIC result, after applying the same correction, becomes 0.27eV. The temperature of the measured molecules is high so this represents an overestimate of the correction (in the high temperature limit, there is no zero-point energy correction).

Supplementary Note 4: Analysis of the Binding Energy Spectra

The time-resolved photoelectron spectra were taken at various pump photon wavelengths are shown in Supplementary Fig. 2. For wavelengths at 216.8 nm and below, the pump pulses resonantly excite DMP to the 3p Rydberg state (Supplementary Figs. 2a to 2d). For longer wavelengths, the 3s Rydberg state is excited (Supplementary Figs. 2e to 2h). The time-delayed probe pulses, at 414.0 nm in the experiment pumping with 207.0 nm and at 404.0 nm in all other cases, monitor the time-dependent dynamics by ionizing the Rydberg-excited molecules. Internal conversion (IC) populates the molecule from the 3p state to 3s within several hundreds of femtoseconds[21]. The 3s states, 3sL and 3sD, with a charge-localized molecular ion core, DMP-L^+, and a charge-delocalized molecular ion core, DMP-D^+, observed at binding energies of 2.81 and 2.70 eV, respectively, transform into each other until an equilibrium is reached after several picoseconds. The IC kinetics and the charge transfer dynamics have been described in detail previously[21].

To analyze the spectral components of the partially overlapping 3s peaks in Supplementary Fig. 2, the spectra were fitted at each delay time with two Lorentzians with variable binding energy centers. The time dependency of the fitted centers is shown in Fig. S3.

Symbols indicate the fitted peak centers at different delay times. Solid lines show the best fits of the centers. For longer pump wavelength (Supplementary Figs. 3e to 3h), data only up to 4 ps was used in the fits because the 3sL peak was too noisy to provide useful center information in the long delay time. The fitted binding energy centers in each experiment and the average center positions are listed in Supplementary Table 1.

Supplementary Note 5: Equilibrium between 3sL and 3sD States

The previous analysis of the BE spectra yields the time dependent fractional intensities of the 3sL and 3sD peaks (symbols in Supplementary Fig. 4). The intensity ratios derived from the deconvoluted peak fits were fitted by adopting the formalism of a two-component equilibrium system that is suddenly displaced from its equilibrium (solid lines in Supplementary Fig. 4)[59]. Assuming equal ionization cross-sections for 3sL and 3sD, and with careful calibration of the intensity sensitivity of the photoelectron spectrometer for electrons with different kinetic energies, the intensity ratio mirrors the population ratio. Therefore, the equilibrium constants for the reaction from 3sL to 3sD can be obtained from the intensity ratio of these two states at equilibrium, as listed in Supplementary Table 2.

Supplementary Note 6: Vibrational Energy in the 3s States

The vibrational energy deposited in the 3s Rydberg excited states was calculated as

$$E_{\text{vib}} = h\nu_{\text{pump}} + E_{\text{b}} - IP_{\text{a}} \tag{6}$$

where $h\nu_{\text{pump}}$ is the energy of the pump photon, E_{b} is the BE of a specific state as obtained from the photoelectron spectra, and IP_{a} is the adiabatic ionization potential (AIP), which was estimated by subtracting the minimum energy of the ground state from the minimum energy of the ion state. The CCSD(T)/Aug-cc-pVDZ calculation gives 7.19 eV for the AIP, which is used in the calculation of the deposited vibrational energy. Since the 3sD peak has a BE of E_{b} = 2.70 eV, the 3sD state therefore has 1.93 eV, 1.71 eV, 1.50 eV, 1.44 eV, 1.12 eV, 1.02 eV, 0.86 eV and 0.66 eV of energy deposited into the vibrational manifolds in the experiments with pump photons at 193.0 nm, 200.0 nm, 207.0 nm, 209.0 nm, 220.8 nm, 224.9 nm, 231.5 nm, and 240.8 nm, respectively. Assuming the energy is distributed across all vibrational modes in accordance with the quantum harmonic oscillator partition functions and with the set of vibrational

frequencies[60] calculated by MP2/Aug-cc-pVDZ using the DMP-D^+ structure and scaled by the factor of 0.959 from NIST[61], the effective vibrational temperatures were estimated as 980 K, 916 K, 854 K, 836 K, 735 K, 700 K, 645 K and 565 K for the 3sD state, respectively. A 4 nm fwhm for the excitation laser pulse was used to estimate the uncertainty of the temperature. A detailed description of the vibrational energy and effective vibrational temperature estimation can be found in the literature[62].

Supplementary Note 7: Entropy Difference between DMP-L^+ and DMP-D^+

Using the calculated vibrational frequencies of the DMP-L^+ and DMP-D^+ obtained with MP2, we estimate the relative entropy of the two states within a harmonic oscillator approximation:

$$\Delta S = S_{DMP-D^+} - S_{DMP-L^+} = R \cdot \ln\left(\frac{\Omega_{DMP-D^+}}{2 \cdot \Omega_{DMP-L^+}} \right) = R \cdot \ln\left(\frac{\prod \nu_{DMP-L^+}}{2 \cdot \prod \nu_{DMP-D^+}} \right) = -26.3 J \cdot K^{-1} \cdot mol^{-1} \qquad (7)$$

where R is the gas constant, Ω is the number of states and ν is the vibrational frequency. The vibrational frequencies were calculated by MP2/Aug-cc-pVDZ using the DMP-L^+ [63] and DMP-D^+ [60] structures and scaled by the factor of 0.959 from NIST[61]. Assuming the vibrations of the molecular ion cores of the 3s Rydberg states mirror those of the ions, one can compare this calculated ΔS value to the experimentally measured entropy change, $\Delta S = -17.7\ (4.6)\ J\cdot K^{-1}\cdot mol^{-1}$, from 3sL to 3sD.

Supplementary References

1. Gaillard, E. R. & Whitten, D. G. Photoinduced electron transfer bond fragmentations. *Acc. Chem. Res.* **29**, 292-297 (1996).
2. Newton, M. D. Quantum chemical probes of electron-transfer kinetics - the nature of donor-acceptor interactions. *Chem. Rev.* **91**, 767-792 (1991).
3. Zewail, A. H. Femtochemistry: Atomic-scale dynamics of the chemical bond using ultrafast lasers - (Nobel lecture). *Angew. Chem. Int. Edit.* **39**, 2587-2631 (2000).
4. Moser, C. C., Keske, J. M., Warncke, K., Farid, R. S. & Dutton, P. L. Nature of biological electron transfer. *Nature* **355**, 796-802 (1992).
5. Sanii, L. & Schuster, G. B. Long-distance charge transport in DNA: Sequence-dependent radical cation injection efficiency. *J. Am. Chem. Soc.* **122**, 11545-11546 (2000).
6. Barnett, R. N., Cleveland, C. L., Joy, A., Landman, U. & Schuster, G. B. Charge migration in DNA: ion-gated transport. *Science* **294**, 567-571 (2001).
7. Frischmann, P. D., Mahata, K. & Wurthner, F. Powering the future of molecular artificial photosynthesis with light-harvesting metallosupramolecular dye assemblies. *Chem. Soc. Rev.* **42**, 1847-1870 (2013).
8. Ge, N. H. *et al.* Femtosecond dynamics of electron localization at interfaces. *Science* **279**, 202-205 (1998).
9. Yeh, A. T., Shank, C. V. & McCusker, J. K. Ultrafast electron localization dynamics following photo-induced charge transfer. *Science* **289**, 935-938 (2000).
10. Bakulin, A. A. *et al.* The role of driving energy and delocalized States for charge separation in organic semiconductors. *Science* **335**, 1340-1344 (2012).
11. Falke, S. M. *et al.* Coherent ultrafast charge transfer in an organic photovoltaic blend. *Science* **344**, 1001-1005 (2014).
12. Najafi, E., Scarborough, T. D., Tang, J. & Zewail, A. Ultrafast dynamics. Four-dimensional imaging of carrier interface dynamics in p-n junctions. *Science* **347**, 164-167 (2015).
13. Cohen, A. J., Mori-Sanchez, P. & Yang, W. T. Insights into current limitations of density functional theory. *Science* **321**, 792-794 (2008).
14. Jónsson, H. Simulation of surface processes. *Proc. Natl. Acad. Sci. U. S. A.* **108**, 944-949 (2011).
15. Baruah, T. & Pederson, M. R. Density functional study on a light-harvesting carotenoid-porphyrin-C_{60} molecular triad. *J. Chem. Phys.* **125**, 164706 (2006).
16. Perdew, J. P. & Zunger, A. Self-interaction correction to density-functional approximations for many-electron systems. *Phys. Rev. B* **23**, 5048-5079 (1981).

17. Pederson, M. R., Heaton, R. A. & Lin, C. C. Local-density Hartree-Fock theory of electronic states of molecules with self-interaction correction. *J. Chem. Phys.* **80**, 1972-1975 (1984).

18. Gudmundsdóttir, H., Zhang, Y., Weber, P. M. & Jónsson, H. Self-interaction corrected density functional calculations of molecular Rydberg states. *J. Chem. Phys.* **139**, 194102 (2013).

19. Lehtola, S. & Jónsson, H. Variational, Self-consistent implementation of the Perdew-Zunger self-interaction correction with complex optimal orbitals. *J. Chem. Theory Comput.* **10**, 5324-5337 (2014); *J. Chem. Theory Comput.* **11**, 5052-5053 (2015).

20. Gudmundsdóttir, H., Jónsson, E. Ö. & Jónsson, H. Calculations of Al dopant in α-quartz using a variational implementation of the Perdew-Zunger self-interaction correction. *New J. Phys.* **17**, 083006 (2015).

21. Deb, S., Cheng, X. & Weber, P. M. Structural dynamics and charge transfer in electronically excited N,N '-Dimethylpiperazine. *J. Phys. Chem. Lett.* **4**, 2780-2784 (2013).

22. Hoffmann, R. Interaction of orbitals through space and through bonds. *Acc. Chem. Res.* **4**, 1-9 (1971).

23. Brouwer, A. M., Langkilde, F. W., Bajdor, K. & Wilbrandt, R. Through-bond interaction in the radical cation of N,N-dimethylpiperazine. Resonance Raman spectroscopy and quantum chemical calculations. *Chem. Phys. Lett.* **225**, 386-390 (1994).

24. Brouwer, A. M. *et al*. Radical cation of N,N-dimethylpiperazine: Dramatic structural effects of orbital interactions through bonds. *J. Am. Chem. Soc.* **120**, 3748-3757 (1998).

25. Gosselin, J. L. & Weber, P. M. Rydberg fingerprint spectroscopy: a new spectroscopic tool with local and global structural sensitivity. *J. Phys. Chem. A* **109**, 4899-4904 (2005).

26. Kuthirummal, N. & Weber, P. M. Rydberg states: sensitive probes of molecular structure. *Chem. Phys. Lett.* **378**, 647-653 (2003).

27. Cheng, X. *et al*. Ultrafast structural dynamics in Rydberg excited N,N,N ',N '-tetramethylethylenediamine: conformation dependent electron lone pair interaction and charge delocalization. *Chem. Sci.* **5**, 4394-4403 (2014).

28. Cheng, X., Zhang, Y., Gao, Y., Jónsson, H. & Weber, P. M. Ultrafast structural pathway of charge transfer in N,N,N',N'-tetramethylethylenediamine. *J. Phys. Chem. A* **119**, 2813-2818 (2015).

29. Gaussian 09, Revision **C.01**, Frisch, M. J. *et al*., Gaussian, Inc., Wallingford CT, 2009.

30. Gaussian 09, Revision **D.01**, Frisch, M. J. *et al*., Gaussian, Inc., Wallingford CT, 2009.

31. Valiev, M. *et al*. NWChem: A comprehensive and scalable open-source solution for large scale molecular simulations. *Comput. Phys. Commun.* **181**, 1477-1489 (2010).

32. Dunning, T. H. Gaussian basis sets for use in correlated molecular calculations. 1. The atoms boron through neon and hydrogen. *J. Chem. Phys.* **90**, 1007-1023 (1989).

33. Kendall, R. A., Dunning, T. H. & Harrison, R. J. Electron affinities of the firstrow atoms revisited. Systematic basis sets and wave functions. *J. Chem. Phys.* **96**, 6796-6806 (1992).

34. Mortensen, J. J., Hansen, L. B. & Jacobsen, K. W. Real-space grid implementation of the projector augmented wave method. *Phys. Rev. B* **71**, 035109 (2005).

35. Enkovaara, J. *et al*. Electronic structure calculations with GPAW: a real-space implementation of the projector augmented wave method. *J. Phys. Condens. Matter* **22**, 253202 (2010).

36. Valdes, A. *et al*. Solar hydrogen production with semiconductor metal oxides: new directions in experiment and theory. *Phys. Chem. Chem. Phys.* **14**, 49-70 (2012).

37. Zhao, Y. & Truhlar, D. G. Benchmark databases for nonbonded interactions and their use to test density functional theory. *J. Chem. Theory Comput.* **1**, 415-432 (2005).

38. Magyar, R. J. & Tretiak, S. Dependence of spurious charge-transfer excited states on orbital exchange in TDDFT: Large molecules and clusters. *J. Chem. Theory Comput.* **3**, 976-987 (2007).

39. Zhao, Y. & Truhlar, D. G. Density functional for spectroscopy: no long-range self-interaction error, good performance for Rydberg and charge-transfer states, and better performance on average than B3LYP for ground states. *J. Phys. Chem. A* **110**, 13126-13130 (2006).

40. Zhao, Y. & Truhlar, D. G. The M06 suite of density functionals for main group thermochemistry, thermochemical kinetics, noncovalent interactions, excited states, and transition elements: two new functionals and systematic testing of four M06-class functionals and 12 other functionals. *Theor. Chem. Acc.* **120**, 215-241 (2008).

41. Zhao, Y. & Truhlar, D. G. Density functionals with broad applicability in chemistry. *Acc. Chem. Res.* **41**, 157-167 (2008).

42. Jónsson, H., Mills, G., & Jacobsen, K. W. Nudged elastic band method for finding minimum energy paths of transitions. In *Classical and Quantum Dynamics in Condensed Phase Simulations* (eds. Berne, B. J., Ciccotti, G. & Coker, D. F.) 385-404 (World Scientific, Singapore, 1998).

43. Gudmundsdóttir, H., Zhang, Y., Weber, P. M. & Jónsson, H. Self-interaction corrected density functional calculations of Rydberg states of molecular clusters: N,N-dimethylisopropylamine. *J. Chem. Phys.* **141**, 234308 (2014).

44. Gavnholt, J., Olsen, T., Engelund, M. & Schiotz, J. Delta self-consistent field method to obtain potential energy surfaces of excited molecules on surfaces. *Phys. Rev. B* **78**, 075441 (2008).

45. Hartree, D. R. The wave mechanics of an atom with a non-Coulomb central field Part I theory and methods. *P. Camb. Philos. Soc.* **24**, 89-110 (1928).

46. Hartree, D. R. The wave mechanics of an atom with a non-Coulomb central field Part II some results and discussion. *P. Camb. Philos. Soc.* **24**, 111-132 (1928).

47. Hartree, D. R. The wave mechanics of an atom with a non-Coulomb central field Part III Term values and intensities in series an optical spectra. *P. Camb. Philos. Soc.* **24**, 426-437 (1928).

48. Klüpfel, S., Klüpfel, P. & Jónsson, H. The effect of the Perdew-Zunger self-interaction correction to density functionals on the energetics of small molecules. *J. Chem. Phys.* **137**, 124102 (2012).

49. Lehtola, S. & Jónsson, H. Unitary optimization of localized molecular orbitals. *J. Chem. Theory Comput.* **9**, 5365-5372 (2013).

50. Klüpfel, S., Klüpfel, P. & Jónsson, H. Importance of complex orbitals in calculating the self-interaction-corrected ground state of atoms. *Phys. Rev. A* **84**, 050501 (2011).

51. Kim, B. J., Thantu, N. & Weber, P. M. High resolution photoelectron spectroscopy: The vibrational spectrum of the 2-aminopyridine cation. *J. Chem. Phys.* **97**, 5384-5391 (1992).

52. Cheng, W. *et al.* Control of local ionization and charge transfer in the bifunctional molecule 2-phenylethyl-N,N-dimethylamine using Rydberg fingerprint spectroscopy. *J. Phys. Chem. A* **109**, 1920-1925 (2005).

53. Perdew, J. P., Burke, K. & Ernzerhof, M. Generalized gradient approximation made simple. *Phys. Rev. Lett.* **77**, 3865-3868 (1996).

54. Kohn, W. & Sham, L. J. Self-consistent equations including exchange and correlation effects. *Phys. Rev.* **140**, A1133-A1138 (1965).

55. Henkelman, G. & Jónsson, H. A climbing-image NEB method for finding saddle points and minimum energy paths. *J. Chem. Phys.* **113**, 9901-9904 (2000).

56. Henkelman, G. & Jónsson, H. Improved tangent estimate in the NEB method for finding minimum energy paths and saddle points. *J. Chem. Phys.* **113**, 9978-9985 (2000).

57. Smidstrup, S., Pedersen, A., Stokbro, K. & Jónsson, H. Improved initial guess for minimum energy path calculations. *J. Chem. Phys.* **140**, 214106 (2014).

58. Melander, M., Laasonen, K. & Jónsson, H. Removing external degrees of freedom from transition state search methods using quaternions. *J. Chem. Phys.* **11**, 1055-1062 (2015).

59. Houston, P.L. *Chemical Kinetics and Reaction Dynamics*, McGraw-Hill, New York, 2001.

60. Vibrational frequencies for DMP-D^+ in cm^{-1}: 129.12, 134.09, 140.44, 180.26, 188.95, 402.94, 404.13, 428.34, 436.01, 450.58, 488.08, 677.03, 752.90, 754.48, 773.73, 811.37,

830.86, 959.43, 975.07, 1014.60, 1017.57, 1040.37, 1055.69, 1083.19, 1105.08, 1130.87, 1137.25, 1209.80, 1221.37, 1229.35, 1237.78, 1284.36, 1297.62, 1305.68, 1321.10, 1337.84, 1373.23, 1397.17, 1404.05, 1406.35, 1417.14, 1424.38, 1426.37, 1428.15, 1439.59, 1877.58, 2893.51, 2902.05, 2958.83, 2960.95, 2962.35, 2983.06, 3017.14, 3017.69, 3042.41, 3043.74, 3047.81, 3049.27, 3069.96, 3070.07.

61. http://cccbdb.nist.gov/vibscalejust.asp.

62. Minitti, M. P., Cardoza, J. D. & Weber, P. M. Rydberg fingerprint spectroscopy of hot molecules: structural dispersion in flexible hydrocarbons. *J. Phys. Chem. A* **110**, 10212-10218 (2006).

63. Vibrational frequencies for DMP-L^+ in cm^{-1}: 38.15, 68.60, 167.03, 193.27, 233.76, 337.23, 349.72, 394.53, 416.32, 443.77, 451.20, 596.17, 730.15, 762.70, 794.19, 879.86, 919.19, 944.18, 985.18, 997.51, 1001.95, 1034.17, 1055.74, 1085.33, 1110.05, 1120.35, 1122.85, 1136.09, 1224.83, 1226.50, 1253.29, 1257.91, 1274.42, 1295.56, 1303.90, 1330.80, 1363.47, 1388.06, 1399.76, 1404.66, 1408.97, 1416.68, 1424.33, 1434.12, 1435.07, 1442.13, 2846.70, 2847.10, 2890.33, 2895.74, 2969.44, 2973.12, 3011.52, 3011.56, 3012.65, 3021.99, 3059.94, 3060.83, 3063.38, 3083.61.
